# Efficacy and safety of intravenous fosphenytoin for patients with acute herpes zoster‐associated pain: A placebo‐controlled randomized trial

**DOI:** 10.1111/1346-8138.17054

**Published:** 2023-12-27

**Authors:** Masako Iseki, Takenobu Yamamoto, Youichi Ogawa, Yuta Majima, Yoichiro Abe, Daisuke Watanabe, Fumimasa Amaya, Toshio Hasegawa, Kazuhiro Inafuku, Toshifumi Kosugi, Yukiko Nomura, Tokiko Deguchi, Toshihisa Hamada, Kenji Shimizu, Saori Arai, Morito Takahashi, Izumi Hamada, Yuko Ishikawa, Makoto Kawashima

**Affiliations:** ^1^ Department of Anesthesiology and Pain Medicine Juntendo University Faculty of Medicine Bunkyo‐ku Tokyo Japan; ^2^ Department of Dermatology Kawasaki Medical School General Medical Center Okayama Okayama Japan; ^3^ Department of Dermatology, Faculty of Medicine University of Yamanashi Chuo Yamanashi Japan; ^4^ Department of Dermatology Shizuoka City Shizuoka Hospital Shizuoka Shizuoka Japan; ^5^ Department of Pain Clinic NTT Medical Center Tokyo Shinagawa Tokyo Japan; ^6^ Department of Dermatology Aichi Medical University School of Medicine Nagakute Aichi Japan; ^7^ Department of Pain Management and Palliative Care Medicine Kyoto Prefectural University of Medicine Kyoto Japan; ^8^ Department of Dermatology and Allergology Juntendo University Shizuoka Hospital Izunokuni Shizuoka Japan; ^9^ Department of Dermatology Kimitsu Chuo Hospital Kisarazu Chiba Japan; ^10^ Department of Palliative Care Saga‐Ken Medical Centre Koseikan Saga Japan; ^11^ Department of Dermatology KKR Sapporo Medical Center Sapporo Hokkaido Japan; ^12^ Division of Dermatology Niigata University Graduate School of Medical and Dental Sciences Niigata Japan; ^13^ Department of Dermatology Takamatsu Red Cross Hospital Takamatsu Kagawa Japan; ^14^ Nobelpharma Co., Ltd. Chuo‐ku Tokyo Japan; ^15^ Tokyo Women's Medical University Shinjuku‐ku Tokyo Japan; ^16^ Present address: Majima Skin Clinic Shizuoka Shizuoka Japan; ^17^ Present address: Department of Dermatology Hakodate Goryoukaku Hospital Hakodate Hokkaido Japan; ^18^ Present address: Futaba Skin Clinic Niigata Niigata Japan; ^19^ Present address: Department of Dermatology International University of Health and Welfare Narita Chiba Japan

**Keywords:** herpes zoster, phenytoin, postherpetic neuralgia, prodrugs, safety

## Abstract

Acute zoster‐associated pain develops in most patients with herpes zoster. Nonopioid analgesics are usually used to treat acute zoster‐associated pain but are frequently ineffective. We administered intravenous fosphenytoin, the prodrug of phenytoin, to patients with acute zoster‐associated pain to examine its analgesic efficacy and safety. At 13 medical institutions in Japan, we conducted a phase II, double‐blind, placebo‐controlled, randomized trial of intravenous fosphenytoin in Japanese inpatients with acute zoster‐associated pain for whom nonopioid analgesics had shown an insufficient analgesic effect. The patients were randomly assigned (1:1:1) to receive a single intravenous dose of fosphenytoin at 18 mg/kg (high dose), a single intravenous dose of fosphenytoin at 12 mg/kg (low dose), or placebo. The primary endpoint was the mean change per hour (slope) in the numerical rating scale score from the baseline score until 120 min after dosing. Seventeen patients were randomly assigned to the low‐dose fosphenytoin group (*n* = 6, median age 62.5 years, range 39–75 years), high‐dose fosphenytoin group (*n* = 5, median age 69.0 years, range 22–75 years), and placebo group (*n* = 5, median age 52.0 years, range 38–72 years). One patient was excluded because of investigational drug dilution failure. This study was discontinued because of the influences of coronavirus disease 2019. The slope was significantly lower in the high‐ and low‐dose fosphenytoin groups than in the placebo group (*P* < 0.001 and *P* = 0.016, respectively). Responsiveness to intravenous fosphenytoin (≥2‐point reduction in the numerical rating scale score from baseline to 120 min after dosing) was inferred at plasma total phenytoin concentrations of 10–15 μg/mL. Treatment‐emergent adverse events caused no safety concerns in the clinical setting and intravenous fosphenytoin was well tolerated. Intravenous fosphenytoin appears to be an effective and promising alternative treatment for acute zoster‐associated pain.

Trial Registration: ClinicalTrials.gov NCT04139330.

## 1 INTRODUCTION

Herpes zoster (HZ) is a common viral disease caused by reactivation of the varicella‐zoster virus, which lies latent in the dorsal root ganglia of the spinal and cranial nerves subsequent to primary varicella‐zoster virus infection.^1^ The clinical course of HZ consists of acute (up to 1 month), subacute, and chronic phases.^2^, ^3^ The acute phase is characterized by a dermatomal skin rash with vesicles, the duration of which is related to the age of the patient and to the dermatomes involved.^4^ Contagion is highest during the phase when the rash is vesicular.^5^ Pain in the acute phase is described as pulsating, shooting, burning, or piercing.^4^ Early attenuation of acute pain may prevent the initiation of central mechanisms of chronic pain, thus, potentially reducing the risk of postherpetic neuralgia (PHN).^6^ Greater acute pain is a risk factor for PHN.^7^

Acute herpetic pain is predominantly nociceptive,^8^ whereas PHN—the chronic pain phase of HZ—is neuropathic.^7^ These two categories of pain, the pathogenic mechanisms of which are considered to differ, are difficult to strictly distinguish from each other and may coexist in some clinical settings. In recent years, this series of pain became collectively termed “zoster‐associated pain” (ZAP).^3^ Nonopioid analgesics (e.g., nonsteroidal anti‐inflammatory drugs [NSAIDs] and acetaminophen) are prescribed as first‐line therapeutic drugs for acute ZAP. However, some patients with severe pain have difficulty achieving pain relief with NSAIDs alone. Therefore, off‐label administration of therapeutic drugs for neuropathic pain, opioid analgesics, and analgesic adjuncts (e.g., antidepressants, antiarrhythmics, and antiepileptics) may be attempted.

Fosphenytoin, the prodrug of the anticonvulsant phenytoin, hinders neuronal depolarization and intra‐axial neurotransmission mainly through its sodium channel‐suppressing activity.^9^, ^10^ Because of this mechanism, phenytoin is considered to suppress not low‐frequency, normal neuronal activity but abnormally high‐frequency, neuronal “firing” only in a specific manner, and it is thus expected to alleviate neuropathic pain caused by HZ. Hatangdi et al.^11^ examined the effects of carbamazepine or phenytoin in combination with antidepressants for the treatment of PHN and obtained satisfactory results in patients receiving a phenytoin–antidepressant combination. Braham and Saia^12^ orally administered phenytoin to two patients with thoracic PHN and reported resolution of the episodes of abrupt, intense pain that had occurred several times a day. Furthermore, Thomas and Muthuswami^13^ orally administered diphenylhydantoin to patients with HZ and reported the suppression of PHN development. To the best of our knowledge, the administration of intravenous fosphenytoin for treatment of acute ZAP has not been reported. However, a rat model of acute thermal nociception demonstrated that peripherally applied anticonvulsants that block voltage‐gated sodium channels (e.g., phenytoin and carbamazepine) or voltage‐gated calcium channels (e.g., gabapentin and ethosuximide) might be used as effective analgesics to alleviate thermal nociception because of their effects on the same cellular targets in the cell bodies of peripheral sensory neurons that convey nociceptive signals to the spinal cord.^14^ Moreover, Takasaki et al.^15^ prepared a mouse HZ pain model to examine the analgesic activity of intravenous fosphenytoin on provoked pain‐like behaviors (allodynia and hyperalgesia) and spontaneous pain‐like behaviors (licking of the affected skin) induced by herpes simplex virus type 1 in mice. The authors confirmed the analgesic effect of intravenous fosphenytoin and considered the drug as a potential option for the treatment of acute HZ pain, especially spontaneous pain.^15^

In the present study, we administered a single intravenous dose of fosphenytoin to patients with acute ZAP to examine its analgesic efficacy and safety.

## 2 METHODS

### 2.1 Study design

A multicenter, collaborative, parallel‐group, randomized, phase II, double‐blind, placebo‐controlled trial was conducted to examine the analgesic efficacy and safety of intravenous fosphenytoin for acute ZAP in patients with HZ. Details of the protocol are provided in Supporting Information S1. The protocol was approved by the institutional review board established at each institution, and the study was conducted in accordance with the Good Clinical Practice guidelines and the ethical principles of the Declaration of Helsinki. Written informed consent was obtained from all patients prior to the trial initiation.

### 2.2 Participants

Patients who met all of the following inclusion criteria were eligible for the study: (1) men or women aged ≥20 years who could be hospitalized; (2) presence of HZ‐associated rash (erythema, papules, blisters, or pustules or combinations thereof); (3) ongoing administration of antivirals and nonopioid analgesics (acetaminophen or NSAIDs) for the treatment of HZ; (4) numerical rating scale (NRS) score of ≥4 points both 120 min before and immediately before administration of intravenous fosphenytoin or placebo; and (5) an increase, no change, or decrease in the NRS score (<2 points) 120 min before dosing of intravenous fosphenytoin or placebo, even after the administration of a nonopioid analgesic. The key exclusion criteria were (1) suspicion of increased intracranial pressure; (2) epilepsy, a severe neuropsychiatric disorder, or impaired consciousness; (3) a malignant tumor, current treatment for human immunodeficiency virus infection, or current treatment with immunosuppressants (however, enrollment of these patients was permitted when they did not have difficulty in activities of daily living and exhibited a good general condition); (4) idiopathic trigeminal neuralgia or other severe pain; and (5) current treatment with opioid analgesics or steroids for relief of acute ZAP.

### 2.3 Interventions

The investigational drugs used in this study were fosphenytoin sodium (75 mg/mL) and a placebo of indistinguishable appearance (physiological saline for injection). Nobelpharma Co., Ltd. (Tokyo, Japan) provided these drugs.

### 2.4 Primary and secondary endpoints

The primary endpoint was the mean change per hour (slope) in the NRS score from baseline to 30, 60, 90, and 120 min after dosing. Lidocaine has proven efficacy in neuropathic pain as well as demonstrating efficacy when pain is assessed after 30 min of intravenous administration in patients with acute ZAP.^16^ In addition, first‐line therapeutic drugs for acute ZAP, such as NSAIDs and acetaminophen, show efficacy within 1 h of administration, therefore immediately after administration or approximately 1 h after administration is considered the critical point to evaluate the immediate efficacy of such drugs. On the basis of previous results, the efficacy of fosphenytoin in reducing the NRS score was evaluated for up to 120 min, including approximately 1 h of intravenous administration, in this study. The secondary endpoints were as follows: (1) the change in the NRS score from baseline to each respective assessment point after dosing; (2) the proportion of responders (defined as patients whose NRS score at 120 min after dosing decreased by ≥2 points relative to the baseline score); (3) change in the quality of life (QOL) score calculated using the EuroQol 5‐Dimension 5‐Level (EQ‐5D‐5L)^17^ at each respective assessment point after dosing; (4) abnormalities in blood pressure, heart rate, respiratory rate, electrocardiographic indices, and oxygen saturation detected via the vital information monitor until 2 h after dosing; and (5) the incidences of treatment‐emergent adverse events (TEAEs) and adverse reactions, including abnormal laboratory changes in hematology, blood chemistry, and urinalysis as well as the incidences of serious TEAEs and serious adverse reactions during the study period. TEAEs were expressed according to the Medical Dictionary for Regulatory Activities (version 24.0)^18^ and were graded in accordance with the Japanese version of the Common Terminology Criteria for Adverse Events (version 4.0).^19^

### 2.5 Sample size

Because the effective dose of fosphenytoin for removal of acute ZAP was unknown when this study was conducted, the feasible and minimum number of patients was established as the number of patients required to assess the efficacy of fosphenytoin. However, the power to detect a significant difference in the primary endpoint between 16 patients in the fosphenytoin group and 16 patients in the placebo group at a two‐sided significance level of 5% was calculated as 86% when presuming that the NRS score at 120 min after dosing would decrease by an average of 2 points (standard deviation 1.8) for the fosphenytoin group compared with that for the placebo group. The required total number of patients was calculated as 48 when pooling the data for the 2 fosphenytoin groups (high‐ and low‐dose) and the placebo group.

### 2.6 Randomization and blinding

Eligible patients were randomly assigned (1:1:1) to the high‐dose fosphenytoin group (18 mg/kg), the low‐dose fosphenytoin group (12 mg/kg), or the placebo group. The maximum dose of fosphenytoin sodium was set at 1200 mg/body. To keep the dosage within that range, patients weighing 67 kg or more in the high‐dose group and 100 kg or more in the low‐dose group were treated at the maximum dose of 1200 mg/body. Patients were assigned to the placebo group in such a manner as to ensure blinding of randomization by providing half of the patients with the same injection volume as in the high‐dose fosphenytoin group (fosphenytoin sodium at 0.24 mL/kg) and the other half of the patients with the same injection volume as in the low‐dose fosphenytoin group (fosphenytoin sodium at 0.16 mL/kg). An independent statistician generated the codes for permuted‐block (size of six) randomization and entered them into a web‐based electronic data capture (EDC) system (DDworks21/EDC plus versions V01.32.00R20190515–V01.33.00R20191108; Fujitsu Limited, Tokyo, Japan). The investigator at each institution administered the investigational drug corresponding to the code allocated by the EDC system to each patient. Patients, investigators, and any other parties concerned, including clinical coordinators, were blinded to the investigational drug allocations (fosphenytoin or placebo group), except dose level (low‐dose or high‐dose in the fosphenytoin and placebo groups), until study completion.

### 2.7 Procedures

The investigational drugs were prepared in a blinded manner in the pharmacy department at each medical institution. The investigational drug allocated to each study group was diluted three‐ to four‐fold with physiological saline for injection and was then set to the infusion pump. The patient received the allocated investigational drug at the bedside for the following durations of time: ≥18 min for patients in the high‐dose fosphenytoin group and the corresponding placebo group, and ≥12 min for patients in the low‐dose fosphenytoin group and the corresponding placebo group. From 2 weeks prior to the start date of dosing to 24 h after dosing, the administration of drugs (e.g., phenytoin, fosphenytoin, ethotoin, and combinations thereof; antiepileptics; calcium channel α2δ ligands; antidepressants; antiarrhythmics; N‐methyl‐D‐aspartate receptor antagonists; central muscle relaxants; local anesthetics; steroids; and opioid analgesics) was prohibited.

### 2.8 Statistical analyses

Regarding the primary endpoint, the between‐group slopes in the (a) high‐dose fosphenytoin group/placebo group and the (b) low‐dose fosphenytoin group/placebo group were compared according to the closed procedure of (a) to (b) when applying a mixed‐effects model that used the random effect (patients) as well as fixed effects (administration group, time of assessment, and administration group × time of assessment [slope]). Regarding the secondary endpoints, a linear regression model using the covariate (the baseline score) and the fixed effect (the administration group) was applied to perform between‐group comparisons with respect to changes in the NRS score at respective assessment points after dosing. In addition, the two‐sided 95% confidence intervals (CIs) for the means were calculated as required. The proportions of responders were examined according to Fisher's exact test, and changes in the QOL score calculated based on the EQ‐5D‐5L were examined according to the Wilcoxon rank‐sum test. The relationships between changes in the NRS score from baseline and the plasma total phenytoin concentration at 120 min after dosing were inferred using the Hamiltonian Monte Carlo method based on a hierarchical Bayesian model that assumed a normal distribution. The virtual NRS scores at four plasma total phenytoin concentrations (0, 5, 10, and 15 μg/mL), each of which comprised 4000 plots, were generated for diagrammatic presentation, and the medians (2.5th to 97.5th percentiles) for changes in the NRS score were estimated. The Spearman's rank correlation coefficient was calculated to test the significance of association between the plasma total phenytoin concentration and change in the NRS score. A *P* value of <0.05 was considered statistically significant. Statistical analyses were conducted using the SAS software package version 9.4 (SAS Institute Inc., Cary, NC, USA) and R version 4.0.2 (The R Foundation for Statistical Computing, Vienna, Austria) for simulation using “brms”^20^ and “ggplot 2 (R package).”

## 3 RESULTS

**Figure 1:**
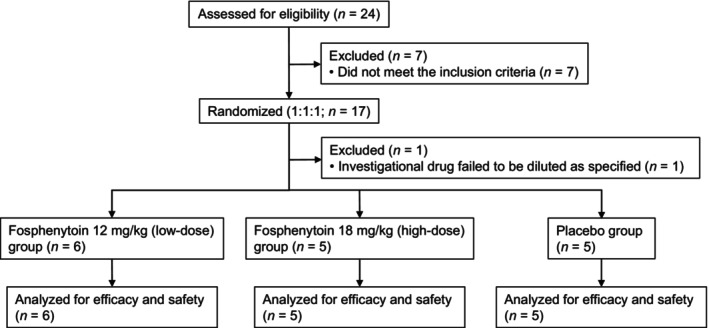
Randomization flow chart. Twenty‐four patients were screened for eligibility, 17 of whom underwent randomization. After excluding one patient for whom the allocated investigational drug was not diluted as specified, 16 patients were analyzed for efficacy and safety and completed the study.

From July 2019 to February 2021, 24 patients were screened for eligibility at 13 medical institutions in Japan, and seven of these patients were excluded because of ineligibility. The remaining 17 patients were randomly assigned (1:1:1). However, one of these remaining 17 patients was excluded before dosing because the allocated drug failed to be diluted as specified. Consequently, 16 patients received low‐dose fosphenytoin (*n* = 6, median age 62.5 years, range 39–75 years), high‐dose fosphenytoin (*n* = 5, median age 69.0 years, range 22–75 years), or placebo (*n* = 5 [2 for the high dose and 3 for the low dose], median age 52.0 years, range 38–72 years) (Figure 1). The present study was initiated with a target sample size of 48 patients but was discontinued halfway because of the influences of coronavirus disease 2019. No great biases were found in the patients' demographic and clinical characteristics among the study groups at baseline (Table 1).

**Table 1:** Demographic and clinical characteristics of the patients at baseline.

Variable	Category	Study group		
		Low dose	High dose	Placebo
*n*		6	5	5
Age (years)	Median	62.5	69.0	52.0
	Range	39–75	22–75	38–72
Sex (*n*)	Male	2	3	3
	Female	4	2	2
Height (cm)	Mean	160.0	161.0	161.6
	SD	9.6	9.7	6.3
Weight (kg)	Mean	68.7	58.7	63.9
	SD	20.0	11.8	9.2
BMI (kg/m^2^)	Mean	26.4	22.5	24.5
	SD	5.7	3.5	3.5
Primary disease				
Location (*n*)	Head to face	4	3	3
	Neck to upper limbs	2	1	0
	Upper limbs to thoracodorsal region	2	3	1
	Buttocks to lower limbs	0	0	1
Type of pain				
Stinging pain (*n*)	No	3	2	3
	Yes	3	3	2
Electric‐like pain (*n*)	No	3	3	5
	Yes	3	2	0
Others (*n*)	No	3	3	1
	Yes	3	2	4

Abbreviations: BMI, body mass index; SD, standard deviation.

**Figure 2:**
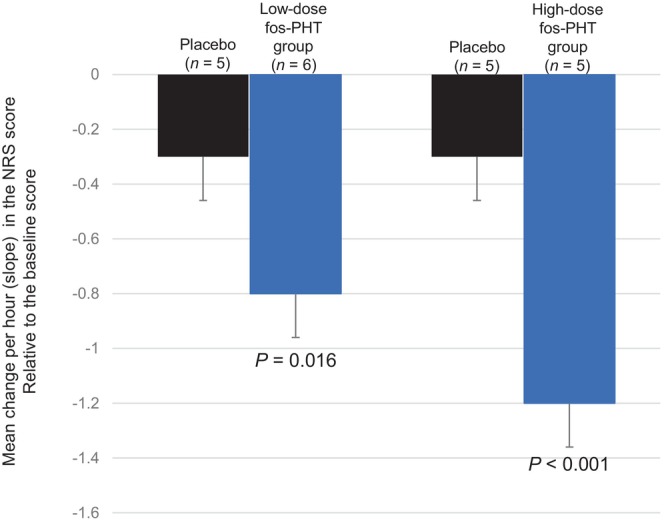
Mean change per hour (slope) in the NRS score from baseline to 120 min after dosing in the low‐ and high‐dose fosphenytoin groups and in the placebo group. The slope was significantly greater in the low‐dose fosphenytoin group than in the placebo group (−0.8 ± 0.14 vs. −0.3 ± 0.15, respectively) and in the high‐dose fosphenytoin group than in the placebo group (−1.2 ± 0.16 vs. −0.3 ± 0.16, respectively). The T bars indicate the standard errors. fos‐PHT, fosphenytoin; NRS, numerical rating scale.

**Figure 3:**
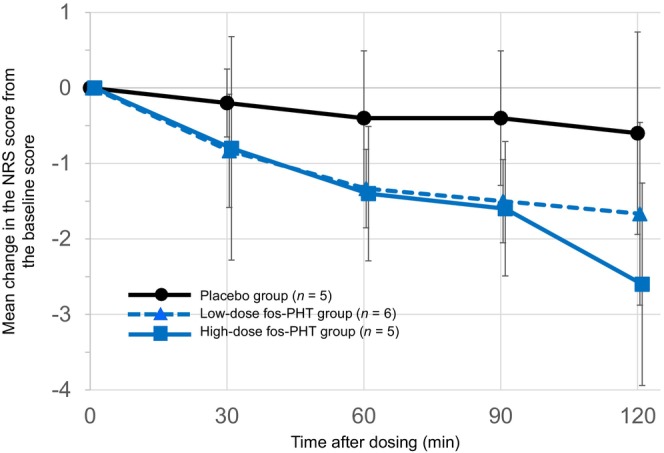
Mean change in the NRS score from baseline to 30, 60, 90, and 120 min after dosing. The T bars indicate the standard deviations. fos‐PHT, fosphenytoin; NRS, numerical rating scale.

The mean changes per hour (slope) in the NRS scores from baseline to 120 min after dosing were −1.2 ± 0.16 and −0.3 ± 0.16 in the high‐dose fosphenytoin group and the placebo group, respectively, with a difference of −0.9 (*P* < 0.001) (Figure 2). The relevant changes were −0.8 ± 0.14 and −0.3 ± 0.15 in the low‐dose fosphenytoin group and the placebo group, respectively, with a difference of −0.5 (*P* = 0.016) (Figure 2). The mean change in the NRS score from baseline to 120 min after dosing tended to decrease in all three study groups. At ≥30 min after dosing, dissociations of the mean changes in the NRS scores were found between the two active drug groups and the placebo group. At 120 min after dosing, the mean changes in the NRS score from baseline in the low‐dose fosphenytoin group, high‐dose fosphenytoin group, and placebo group were −1.7 ± 1.21, −2.6 ± 1.34, and −0.6 ± 1.34, respectively (Figure 3).

The proportions of responders in the low‐dose fosphenytoin group, high‐dose fosphenytoin group, and placebo group were 33.3% (95% CI 4.3–77.7%), 100.0% (95% CI 47.8–100.0%), and 20.0% (95% CI 0.5–71.6%), respectively. A statistically significant difference in this proportion was found between the high‐dose fosphenytoin group and the placebo group (*P* = 0.048), but not between the low‐dose fosphenytoin group and the placebo group (*P* = 1.000). No statistically significant difference was found in the changes in the NRS scores from baseline to ≥4 h after dosing between the two active drug groups and the placebo group. Likewise, no statistically significant difference was found in the QOL score calculated based on the EQ‐5D‐5L at any time point after dosing.

**Figure 4:**
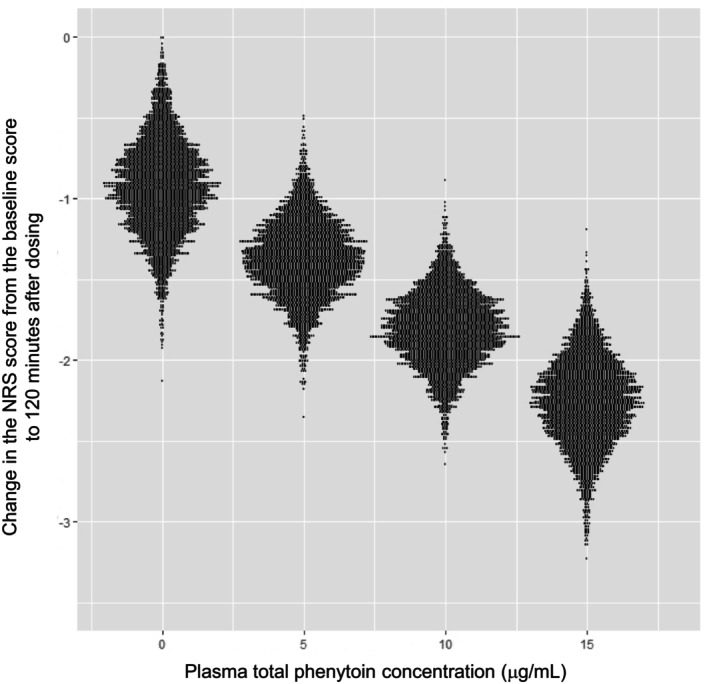
Simulation for the inference of relationships between changes from baseline in the NRS score 120 min after dosing and four plasma total phenytoin concentrations. NRS, numerical rating scale. The Hamiltonian Monte Carlo method based on a hierarchical Bayesian model was used for the simulation; 4000 virtual NRS scores were created at each plasma total phenytoin concentration.

The relationship between the change in the NRS score from baseline to 120 min after dosing and the plasma total phenytoin concentration is shown in Figure 4. The median (2.5th–97.5th percentiles) changes in the NRS score from baseline to 120 min after dosing were estimated to be −0.93 (−1.49 to −0.33), −1.37 (−1.83 to −0.90), −1.81 (−2.26 to −1.37), and −2.25 (−2.79 to −1.72) at plasma total phenytoin concentrations of 0, 5, 10, and 15 μg/mL, respectively. Significant correlation was found between plasma total phenytoin concentration and change in the NRS score (Spearman's rank correlation *ρ* = −0.693 [*P* < 0.001]). These results suggest concentration‐dependent changes in the NRS score.

No deaths or serious TEAEs occurred in this study; all TEAEs were grade 1 or 2 in severity. Adverse drug reactions were experienced in three out of six patients (50%) in the low‐dose fosphenytoin group, three out of five patients (60%) in the high‐dose fosphenytoin group, and zero out of five patients (0%) in the placebo group. All events were grade 1 in severity. Intravenous fosphenytoin did not cause any safety concerns in the clinical setting because all TEAEs were manageable with or without medication (Table 2).

**Table 2:** Treatment‐emergent adverse events.

Institution no.‐patient no.	Group	Preferred term	Time after dosing (h)	Grade^a^	Causality
01‐01	High‐dose^b^	Vertigo	0.58	1	Related
		Tinnitus	0.58	1	Related
03‐02	High‐dose	Tinnitus	0.58	1	Related
03‐05	Placebo	Dizziness	28.72	1	Not related
		Urinary retention	49.60	2	Not related
		Constipation	75.27	2	Not related
		Dermatitis	75.30	2	Not related
03‐06	Low‐dose^c^	Tinnitus	0.50	1	Related
		Liver function test result(s) increased^d^	23.95	1	Not related
		Drug eruption	57.92	2	Not related
05‐01	High‐dose	Nausea	0.25	1	Related
		Dizziness	2.00	1	Related
05‐08	Low‐dose	Blood pressure decreased	0.50	1	Related
		Pyrexia	7.18	1	Related
10‐01	Placebo	Pyrexia	4.20	1	Not related
		Lymphocyte morphology abnormal	23.10	1	Not related
		Blood uric acid increased	138.60	1	Not related
		Total protein abnormal	138.60	1	Not related
		Blood albumin decreased	138.60	1	Not related
		Hepatic function abnormal	138.60	1	Not related
10‐02	Low‐dose	Somnolence	0.60	1	Related
		Pruritus	31.93	2	Not related

^a^ Common Terminology Criteria for Adverse Events (version 4.0).

^b^ Fosphenytoin (18 mg/kg).

^c^ Fosphenytoin (12 mg/kg).

^d^ Increases in aspartate aminotransferase and alanine aminotransferase concentrations.

## 4 DISCUSSION

Acute ZAP is intolerable in many patients with HZ and requires therapeutic interventions. Moderate to severe ZAP occurs in 60%–70% of patients with HZ, resulting in impaired activities of daily living and reductions in emotional, physical, and social functioning.^3^ Patients with HZ usually undergo pharmacotherapy with nonopioid analgesics. However, the analgesic effect of these drugs is insufficient in some clinical settings. The antiepileptic gabapentin is used as an analgesic adjunct for neuropathic pain. In one study, a single dose of oral gabapentin caused a 66% reduction in the visual analogue scale (VAS)‐assessed intensity of acute ZAP compared with a 33% reduction attained with placebo.^21^ Additionally, a single oral dose of pregabalin, a therapeutic drug for neuropathic pain, reduced the VAS‐assessed intensity of acute ZAP by a mean of 33% compared with placebo, which reduced it by a mean of 14%.^22^ The lidocaine patch (not approved in Japan), which is used for the treatment of PHN in the United States and Europe, significantly reduced the VAS‐assessed intensity of acute pain at rest and during movement (14.7 [*P* = 0.005] and 10.4 [*P* = 0.007], respectively) compared with placebo.^23^ All of these drugs are frequently used, often in an off‐label manner, in real‐world clinical settings to treat patients with acute ZAP.

Despite the limited number of patients, the present study also showed that compared with placebo, a single dose of intravenous fosphenytoin exerted a significant analgesic effect in patients with acute ZAP for which nonopioid analgesics had been insufficient.

Forbes et al.^24^ conducted a meta‐analysis of risk factors for PHN and found an association between acute pain and the risk of PHN (rate ratio 2.23, 95% CI 1.71–2.92, *P*_heterogeneity_ = 0.649, *I* ^2^ = 0.0%) in eight cohort studies reporting severe acute pain as a binary variable that enabled estimate pooling. These findings indicate that the removal of acute ZAP not only improves the QOL of patients with HZ for a short period of time but also holds promise for reducing the risk of difficult‐to‐treat complications caused by PHN.

Considering that pregabalin caused a 33% reduction in the VAS‐assessed intensity of acute ZAP and that the lidocaine patch caused reductions in the VAS‐assessed pain intensity during rest and movement by 28 and 24 points, respectively, from the baseline scores, we conjecture that the analgesic effect of a single dose of intravenous fosphenytoin is not inferior to that of pregabalin or lidocaine.

A single high dose of intravenous fosphenytoin had a stronger analgesic effect, as shown by greater decreases in the NRS scores than those achieved with low‐dose fosphenytoin. The ad hoc analysis according to the Hamiltonian Monte Carlo method based on a hierarchical Bayesian model revealed responsiveness (≥2‐point reduction in the NRS score at 120 min after dosing relative to the baseline score) to a single dose of intravenous fosphenytoin at a plasma total phenytoin concentration of 10–15 μg/mL. When used as an antiepileptic, fosphenytoin reaches the therapeutic window (plasma total phenytoin concentration of 10–20 μg/mL).^25^ Of interest is the finding that fosphenytoin reaches similar therapeutic windows when used as an analgesic adjunct and an antiepileptic.

Neither deaths nor serious TEAEs occurred during the present study, and all TEAEs that occurred were grade 1 or 2 in severity, therefore intravenous fosphenytoin was safe and well tolerated, and did not cause any clinical concerns because all TEAEs were manageable with or without medication.

### 4.1 Limitations

The present study has several limitations. First, the number of patients examined was limited, thus caution is required when interpreting the study results. Second, the duration of the analgesic effect (>2 h) of intravenous fosphenytoin given by a single dose requires further investigation. Third, the analgesic effect and safety of intravenous fosphenytoin given by repeated administration need to be verified.

## 5 CONCLUSION

The present study revealed a rapid analgesic effect of a single dose of intravenous fosphenytoin for acute ZAP in patients with HZ for whom nonopioid drugs had shown an insufficient analgesic effect. Therefore, intravenous fosphenytoin holds promise as a potential alternative treatment for acute ZAP. A further confirmatory clinical study is required.

## CONFLICT OF INTEREST STATEMENT

M.I., T.Y., Y.O., Y.M., Y.A., D.W., F.A., T.H., K.I., T.K., Y.N., T.D., T.H., and M.K. have received article processing fees form Nobelpharma Co., Ltd. M.I. has received consulting fees from Nobelpharma Co., Ltd. as well as honoraria for lectures from Daiichi Sankyo Co., Ltd., Shionogi & Co., Ltd., Pfizer Inc., and Viatris Inc. M.I. has leadership roles in the Japan Society of Pain Clinicians, the Japanese Society for the Study of Chronic Pain, and the Japanese Association for the Study of Pain. D.W. has received honoraria for lectures from Maruho Co., Ltd., Takeda Pharmaceutical Co., Ltd., GlaxoSmithKline plc., Daiichi Sankyo Co., Ltd., and Taiho Pharmaceutical Co., Ltd., and has received support for attending meetings from GlaxoSmithKline plc. D.W. has participated in the Advisory Board of Maruho Co., Ltd. D.W. has leadership roles in the Japanese Dermatological Association and the Japanese Society for Sexually Transmitted Infections. T.K. has received payment for lectures from Daiichi Sankyo Co., Ltd. and Hisamitsu Pharmaceutical Co., Inc. M.K. has received consulting fees from Nobelpharma Co., Ltd. K.S., S.A., M.T., I.H., and Y.I. are employees of Nobelpharma Co., Ltd.

## Supporting information


Supporting Information Data S1.
