# Supplementary material for: Efficacy and safety of intravenous fosphenytoin for patients with acute herpes zoster‐associated pain: A placebo‐controlled randomized trial
Source: J Dermatol. 2023 Dec 27;51(2):234–42. doi: 10.1111/1346-8138.17054 (PMC11484135; doi:10.1111/1346-8138.17054)
Supplement: Supplementary file 1 — Supporting Information Data S1. [file JDE-51--s001.docx]

A Phase II Placebo-controlled, Double-blind, Comparative Study of NPC-06 in Patients with Pain Associated with Acute Herpes Zoster

Clinical Study Protocol

Sponsor: Nobelpharma Co., Ltd.

Clinical study protocol No.: NPC-06-5

Version No.: Ver. 4.00

Date of preparation: December 3, 2020

[Confidentiality Statement]

This clinical study protocol is copyrighted by Nobelpharma Co., Ltd. under the Copyright Act. Copying, reproducing, or reprinting this clinical study protocol, in whole or in part, is prohibited in any way, including photocopying or input into a magnetic or optical medium.

The information contained in this document (particularly unpublished data) is confidential information and provided only to the director, investigator, subinvestigators (hereinafter referred to the investigators), clinical research coordinators, the institutional review board (IRB), and investigational product administrator at study sites that will or are willing to participate in the study. Any publication or disclosure of this clinical study protocol to any third party requires the prior written approval of Nobelpharma Co., Ltd.

# CLINICAL STUDY PROTOCOL SYNOPSIS

| Study title | A Phase II placebo-controlled, double-blind, comparative study of NPC-06 in patients with pain associated with acute herpes zoster |
| --- | --- |
| Clinical study protocol No. | NPC-06-5 |
| Objective of the study | This study is intended to examine the pain-relieving effect and safety of NPC-06 (fosphenytoin sodium injection) in patients with pain associated with acute herpes zoster in a placebo-controlled, double-blind, parallel-group, comparative manner. This study is also intended to explore the effective concentration range of NPC-06. |
| Study design | Multicenter, randomized, placebo-controlled, double-blind, parallel-group, comparative study |
| Phase of development | Phase II exploratory study |
| Patient population | Patients with pain associated with acute herpes zoster |
| Inclusion criteria | Patients who meet all the following criteria at the subject registration will be included.   1. Patients aged 20 years or older at the acquisition of informed consent 2. Sex: Either male or female 3. Patients with herpes zoster who present with acute onset of skin rash (single or concurrent occurrence of erythema, papule, blister, and pustule). However, patients who have crust formation of more than 20% of the whole rash immediately before the start of the study treatment will be excluded. 4. Patients who are on an antiviral drug for herpes zoster at the start of the study treatment 5. Patients who are on a non-opioid analgesic (acetaminophen or NSAIDs) for herpes zoster at the start of the study treatment. 6. Patients with pain intensity of ≥ 4 NRS score at the following evaluation points   - 120 minutes before the start of the study treatment  - Immediately before the start of the study treatment   1. Patients with poor response to non-opioid analgesics for herpes zoster   The term “poor response” is defined as a condition that meets both of the following criteria.  - The mean pain intensity is ≥ 4 NRS score in spite of the treatment with non-opioid analgesics from 24 hours to 120 minutes before the start of the study treatment (non-opioid analgesics are used at least twice during the period).  - The reduction in NRS score at immediately before the start of the study treatment (120 minutes after the administration of non-opioid analgesics) is less than 2 points in comparison with the score at 120 minutes before the start of the study treatment (before the administration of non-opioid analgesics) despite the treatment with non-opioid analgesics before the study treatment.   1. Patients who can be hospitalized 2. Patients for whom written informed consent was obtained with the patient’s own or legally acceptable representative’s free will with full understanding after receiving sufficient explanation. In the case where a legally acceptable representative is required for the patient, written informed consent must be obtained from both the patient and his or her legally acceptable representative. |
| Exclusion criteria | Patients who meet any of the following criteria at the subject registration will be excluded.   1. Patients who cannot evaluate pain with NRS 2. Patients suspected to have increased intracranial pressure 3. Patients complicated with epilepsy, serious neuropsychiatric disorders (e.g., dementia, Parkinson's disease, or schizophrenia), or consciousness disturbance 4. Patients complicated with malignant tumors (excluding patients who are under treatment management and show no sign of recurrence), patients being treated for HIV, and patients being treated with immunosuppressants (including biologics) (excluding patients who have no hindrance in daily life and whose general condition is favorable) 5. Patients complicated with idiopathic trigeminal neuralgia 6. Patients with other severe pain that may affect the assessment of pain associated with acute herpes zoster 7. Patients being treated with opioid analgesics or steroid anti-inflammatory drugs (systemic) for analgesia of pain associated with acute herpes zoster 8. Patients with sinus bradycardia or advanced conduction disturbance 9. Patients with a history of hypersensitivity to hydantoin drugs 10. Patients being treated with tadalafil (for pulmonary hypertension), rilpivirine hydrochloride, asunaprevir, daclatasvir hydrochloride, macitentan, elbasvir, grazoprevir hydrate, ticagrelor, artemether・lumefantrine, daclatasvir hydrochloride・asunaprevir・beclabuvir hydrochloride, darunavir ethanolate・cobicistat, rilpivirine hydrochloride・tenofovir disoproxil fumarate・emtricitabine, rilpivirine hydrochloride・tenofovir alafenamide fumarate・emtricitabine, bictegravir sodium・emtricitabine・tenofovir alafenamide fumarate, elvitegravir・cobicistat・emtricitabine・tenofovir alafenamide fumarate, elvitegravir・cobicistat・emtricitabine・tenofovir disoproxil fumarate, sofosbuvir・velpatasvir, sofosbuvir, ledipasvir acetonate・sofosbuvi, or dolutegravir sodium・rilpivirine hydrochloride 11. Patients being treated with drugs for neuropathic pain, antidepressants, antiarrhythmics, NMDA receptor antagonists, central muscle relaxants, local anesthetics, vaccinia virus inoculated rabbit inflamed skin extract, Chinese herbal medicines and vitamin B_12_ preparations for analgesia of the pain associated with acute herpes zoster 12. Patients treated with amenamevir within 24 hours before the start of the study treatment 13. Patients complicated with meningitis or patients with meningeal irritation symptoms 14. Patients with a serious cardiac disease, respiratory disorder, or hepatic/renal impairment (the term “serious” refers to Grade 3 in the Standards for Classification of Seriousness of Adverse Drug Reactions by Drugs etc. (PAB/SD Notification No. 80)) 15. Patients being treated with fosphenytoin, phenytoin, ethotoin, or any combination drug containing any of these drugs, or patients who received any of these drugs as an analgesic adjuvant 16. Patients who participated in another clinical trial within 3 months before the date of the screening test 17. Pregnant or lactating women or patients who may become pregnant during the study period 18. Patients unable to agree to use appropriate contraception as instructed by the investigator or subinvestigator (hereinafter referred to as the investigators) during the period from the acquisition of informed consent to the end of the post-treatment observation period 19. Other patients inappropriate for the participation in this study, as judged by the investigators |
| Investigational products | 1. Test drug: NPC-06   Colorless to pale yellow, clear solution for injection containing 750 mg of fosphenytoin sodium per 10 mL in a vial   1. Control drug: Japanese Pharmacopoeia (JP) Isotonic Sodium Chloride Solution   Ten mL of isotonic sodium chloride solution in a vial indistinguishable from the test drug by appearance (clear and colorless) |
| Dosage and administration | [High-dose group]  <Dose>  Eighteen mg/kg of NPC-06 or placebo will be administered once by intravenous drip infusion.  <Administration method>  The investigational product will be diluted 3 to 4-fold with a physiological saline solution for drip infusion and infused over 18 minutes or longer.  [Low-dose group]  <Dose>  Twelve mg/kg of NPC-06 or placebo will be administered once by intravenous drip infusion.  <Administration method>  The investigational product will be diluted 3 to 4-fold with a physiological saline solution for drip infusion and infused over 12 minutes or longer.  In this regard, however, the dosage of NPC-06 must not exceed 1,200 mg on the basis of fosphenytoin sodium in both dose groups.  For convenience, subjects treated with NPC-06 in the high-dose and low-dose groups are hereinafter referred to as the high-dose group and the low-dose group, respectively. Subjects treated with placebo are referred to as the placebo group. |
| Administration period  Observation period | Administration period: Single-dose administration  Hospitalization period: 2 to 3 days Post-treatment observation period: 6 days after the next day of administration ± 2 days |
| Prohibited concomitant drugs/therapies | (1) Prohibited concomitant drugs  <Prohibited from 2 weeks before the start of the study treatment to the end of the evaluation at 24 hours after administration on the next day of the study treatment (Day 2)>  - Phenytoin, fosphenytoin, ethotoin, and combination drugs containing any of these drugs  - Antiepileptic drugs, tadalafil, rilpivirine hydrochloride, asunaprevir, daclatasvir hydrochloride, macitentan, elbasvir, grazoprevir hydrate, ticagrelor, artemether・lumefantrine, daclatasvir hydrochloride・asunaprevir・beclabuvir hydrochloride, darunavir ethanolate・cobicistat, rilpivirine hydrochloride・tenofovir disoproxil fumarate・emtricitabine, rilpivirine hydrochloride・tenofovir alafenamide fumarate・emtricitabine, bictegravir sodium・emtricitabine・tenofovir alafenamide fumarate, elvitegravir・cobicistat・emtricitabine・tenofovir alafenamide fumarate, elvitegravir・cobicistat・emtricitabine・tenofovir disoproxil fumarate, sofosbuvir・velpatasvir, sofosbuvir, ledipasvir acetonate・sofosbuvi, or dolutegravir sodium・rilpivirine hydrochloride  - Drugs for neuropathic pain, antidepressants, antiarrhythmics, NMDA receptor antagonists, central muscle relaxants, and local anesthetics  - Opioid analgesics  - Steroidal anti-inflammatory drugs (systemic) for analgesia of pain associated with acute herpes zoster  - Vaccinia virus inoculated rabbit inflamed skin extract  - Chinese herbal medicines for analgesia of pain associated with acute herpes zoster  - Vitamin B_12_ preparations for analgesia of pain associated with acute herpes zoster  <Prohibited from 24 hours before the start of the study treatment to the end of the evaluation at 24 hours after administration on the next day of the study treatment (Day 2)>  - Amenamevir  - Sustained release non-opioid analgesics  (2) Prohibited concomitant therapies  <Prohibited from the onset of herpes zoster to the end of the evaluation at 24 hours after administration on the next day of the study treatment (Day 2)>  - Nerve block therapy for the efficacy evaluation site  - Anti-inflammatory analgesic treatment (e.g., low-power laser therapy, low-power ultrasonic therapy)  - Other surgical treatments for pain  (3) Restricted concomitant drugs  <The switch to another drug, change in the route of administration or dosage and administration of the current drugs, or addition of a new drug is prohibited from the onset of herpes zoster to the end of the evaluation at 24 hours after administration on the next day of the study treatment (Day 2)>  - Aspirin preparations for antiplatelet action  - Steroidal anti-inflammatory drugs (systemic)  - Vitamin B_12_ preparations  - Immunosuppressants (including biologics) |
| Efficacy endpoints | The following endpoints will be compared between each dose and placebo group.   1. Primary endpoint 2. Slope in the NRS score over the evaluation period (mean score change) based on the NRS score before (baseline) and 30, 60, 90, and 120 minutes after the start of the study treatment 3. Secondary endpoints 4. Change in the NRS score at each evaluation point from the baseline 5. Proportion of patients in whom the NRS score is improved by at least 2 points at 120 minutes after the start of the study treatment as compared to immediately before the start of the study treatment 6. Change in QOL value of EQ-5D-5L 7. Other endpoints 8. Comparison between each dose and placebo group 9. Relationship between blood total phenytoin concentration and NRS score |
| Safety endpoints | The safety profile of the following endpoints will be examined among the groups (high-dose group, low-dose group, and placebo group).   1. Incidence of adverse events and adverse drug reactions 2. Incidence of serious adverse events and adverse drug reactions and narratives of individual events 3. Laboratory tests (hematology, blood biochemistry, and urinalysis) 4. Incidence of abnormal findings of blood pressure, heart rate, respiratory rate, electrocardiogram, and SpO_2_, as measured with biological information monitor |
| Target number of subjects | 48 subjects (16 subjects in the high-dose group, 16 subjects in the low dose group, and 16 subjects in the placebo group) |
| Medical expert | Masako Iseki, Professor, Department of Anesthesiology and Pain Medicine, Juntendo University School of Medicine |
| Planned study period | June 2019 to March 2021 |

Study Schedule

| Timing  Item | | | Screening ^*1^ | Investigational product administration day | | | | | | | | | | | Post-treatment observation period | | | | | Day of discontinuation |
| --- | --- | --- | --- | --- | --- | --- | --- | --- | --- | --- | --- | --- | --- | --- | --- | --- | --- | --- | --- | --- |
|  |  |  |  | Day 1 (day of administration) | | | | | | | | | | | Day 2 | | | Da y3 to Day 6 | Day7 |  |
|  |  |  |  | 120 minutes before the  start of administration | Immediately before the  start of administration | End of administration | After the start of administration | | | | | | | At bedtime | At the time of awakening | 24 hours after the start of administration | At bedtime | At bedtime | 6 days after the start of administration | At discontinuation |
|  |  |  |  | Before the  administration  of non-opioid  analgesics | 120 minutes  after the  administrationof non-opioid  analgesics |  | 30 minutes | 60 minutes | 90 minutes | 120 minutes | 4 hours | 6 hours | 8 hours |  |  |  |  |  |  |  |
| Acceptable time window^※1^ | | | On and after the day before administration | ± 10 minutes | -^※2^ | - | ± 5 minutes | ± 10 minutes | ± 10 minutes | ± 10 minutes | ± 15 minutes | ± 15 minutes | ± 30 minutes | - | - | ± 3 hours | - | - | ± 2 days | - |
| Informed consent ^*2^ | | | ● |  |  |  |  |  |  |  |  |  |  |  |  |  |  |  |  |  |
| Eligibility check | | | ● |  | ● |  |  |  |  |  |  |  |  |  |  |  |  |  |  |  |
| Subject registration | | |  |  | ● |  |  |  |  |  |  |  |  |  |  |  |  |  |  |  |
| Demographic and other characteristics | | | ● |  |  |  |  |  |  |  |  |  |  |  |  |  |  |  |  |  |
| Medical examination | | | ● | ● | ● |  |  |  |  | ● |  |  |  |  |  | ● |  |  | ● | ● |
| Height and body weight | | | ● |  |  |  |  |  |  |  |  |  |  |  |  |  |  |  |  |  |
| Vital signs | | | ● |  |  |  |  |  |  |  |  |  |  |  |  | ● |  |  | ● | ● |
| Biological information monitor ^*3^ | | |  |  |  |  |  |  |  |  |  |  |  |  |  |  |  |  |  |  |
| Study treatment^*4^ | | |  |  |  |  |  |  |  |  |  |  |  |  |  |  |  |  |  |  |
| Administration of non-opioid analgesics^*5^ | | | ● | ● |  |  |  |  |  |  |  |  |  |  |  |  |  |  |  |  |
| Patient diary  Efficacy evaluation | NRS | Evaluation point^*6^ | ● | ● | ● |  | ● | ● | ● | ● | ● | ● | ●^*8^ | ●^*9^ | ● | ● |  |  | ● | ●^*12^ |
|  |  | Mean^*7^ |  | ● |  |  |  |  |  |  |  |  |  |  |  |  |  |  |  |  |
|  |  | Maximum |  |  |  |  |  |  |  |  |  |  |  |  |  |  | ●^*10^ | ●^*11^ |  |  |
|  | EQ-5D-5L | | ● |  | ● |  |  |  |  | ● |  |  |  | ● |  | ● | ● | ● | ● | ●^*12^ |
| Laboratory tests | | | ●^*13^ |  |  |  |  |  |  |  |  |  |  |  |  | ● |  |  | ● | ● |
| Pregnancy test ^*14^ | | | ● |  |  |  |  |  |  |  |  |  |  |  |  |  |  |  | ● | ● |
| Blood drug concentration | | | ● |  |  |  | ● |  |  | ● |  |  |  |  |  | ● |  |  |  |  |
| Adverse events | | |  |  |  |  |  |  |  |  |  |  |  |  |  |  |  |  |  | ● |
| Concomitant drugs/therapies | | |  |  |  |  |  |  |  |  |  |  |  |  |  |  |  |  |  | ● |

※1: The starting point of the acceptable time window after the study treatment is the start time of administration.

※2: Perform evaluation and observation items at 120 minutes ± 10 minutes after the administration time of non-opioid analgesics and perform subject registration.

*1: Perform screening on the day before administration or at least 120 minutes before the start of the study treatment on Day 1 (before the administration of non-opiod analgesics).

*2: Obtain written consent before starting the screening test.

*3: Continuously monitor blood pressure, heart rate, respiratory rate, electrocardiogram, and SpO_2_ on the biological information monitor.

*4: Start administering the investigational product within 60 minutes after subject registration as a rough guide and by 15:00 on Day 1 (day of administration).

*5: In principle, use the same non-opioid analgesic(s) during the period from the screening test to the end of evaluation at 24 hours after the start of the study treatment on Day 2.

*6: Record the pain intensity at the evaluation point using the NRS.

*7: Record the mean pain intensity over the past 24 hours using the NRS.

*8: Perform the evaluation at 8 hours after the start of administration as much as possible.

*9: If pain worsens after bedtime on Day 1, evaluate it according to the NRS and investigate the drug(s) used.
Subjects who waken at night for reasons other than the worsening of pain should evaluate the cause according to the NRS as much as possible.

*10: At bedtime on Day 2: Record the maximum pain intensity after the evaluation at 24 hours after the start of the study treatment using the NRS.

*11: At bedtime from Day 3 to Day 6: Record the maximum pain intensity over the past 24 hours using the NRS.

*12: Perform only when discontinued within 24 hours after the start of administration.

*13: If laboratory tests are performed by 3 days before Day 1, the data may be adopted.

*14: To be performed only for women of childbearing potential.

# LIST OF ABBREVIATIONS AND DEFINITION OF TERMS

| Term | English |
| --- | --- |
| ADL | Activities of Daily Living |
| CDISC | Clinical Data Interchange Standards Consortium |
| EDC | Electronic Data Capture |
| EQ-5D-5L | EQ-5D-5L |
| FAS | Full Analysis Set |
| GCP | Good Clinical Practice |
| NRS | Numerical Rating Scale |
| NSAIDs | Non-Steroidal Anti-Inflammatory Drugs |
| MedDRA | the Medical Dictionary for Regulatory Activities |
| PPS | Per Protocol Set |
| SP | Safety Population |
| VZV | Varicella Zoster Virus |

Table of Contents

CLINICAL STUDY PROTOCOL SYNOPSIS i

LIST OF ABBREVIATIONS AND DEFINITION OF TERMS viii

1 HISTORY AND BACKGROUND OF DEVELOPMENT 5

1.1 Overview of Investigational Products 5

1.1.1 Physical, chemical, and pharmaceutical properties and formulation 5

1.1.2 Pharmacology 5

1.1.3 Pharmacokinetics in animals 6

1.1.4 Toxicity 6

1.1.5 Clinical pharmacokinetics 9

1.1.6 Clinical efficacy 10

1.1.7 Clinical safety 10

1.2 History of Development 11

2 OBJECTIVE 12

2.1 Objective of the Study 12

2.2 Phase of Development 12

3 STUDY DESIGN 12

3.1 Study Design 12

3.2 Group Structure 12

3.3 Dosage and Administration Method 12

3.4 Target Number of Subjects 14

3.5 Study Period 14

4 PATIENT POPULATION 15

4.1 Target Disease 15

4.2 Inclusion Criteria 15

4.3 Exclusion Criteria 16

5 REGISTRATION OF SUBJECTS 18

5.1 Preparation of Subject Screening and Subject Registration Lists 18

5.2 Registration Procedure 18

5.3 Preparation of Investigational Product Assignment Table, Blinding Method, and Maintenance of Blinding 19

5.3.1 Storage of investigational product assignment table and emergency code 19

5.3.2 Unblinding procedure 19

5.3.3 Unblinding procedure during the study 19

6 INVESTIGATIONAL PRODUCTS 20

6.1 Name and Other Information of Investigational Products 20

6.2 Components/content and Dosage Form 20

6.2.1 Packaging form 21

6.2.2 Labeling 21

6.3 Control of Investigational Products 21

6.3.1 Storage 21

6.3.2 Shelf life 21

6.3.3 Delivery 21

6.3.4 Storage and control 22

6.3.5 Collection 22

7 ADMINISTRATION OF THE INVESTIGATIONAL PRODUCTS AND MONITORING OF SUBJECTS 22

7.1 Dosage and Administration Method of the Investigational Products 22

7.2 Preparation of Investigational Products 23

7.3 Monitoring of Subjects 24

8 CONCOMITANT DRUGS, CONCOMITANT THERAPIES, AND OTHER RESTRICTIONS 24

8.1 Prohibited Concomitant Drugs 24

8.2 Prohibited Concomitant Therapies 25

8.3 Restricted Concomitant Drugs 25

8.4 Other Restrictions 25

9 ENDPOINTS 26

9.1 Primary Endpoint 26

9.2 Secondary Endpoints 26

9.3 Other Endpoints 27

9.4 Safety Endpoints 27

10 OBSERVATION/EXAMINATION/EVALUATION ITEMS AND TIMING 28

10.1 Observation, Examination, and Evaluation Items at Screening 31

10.2 Observation, Examination, and Evaluation Items during the Study Treatment Period 31

10.2.1 Day of administration (Day 1) 31

10.3 Observation, Examination, and Evaluation Items during the Post-treatment Observation Period 35

10.3.1 Next day of the administration of the investigational product (Day 2) 35

10.3.2 Two days after administration (Day 3) or later 36

10.3.3 Six days after the start of administration of the investigational product (Day 7) 36

10.3.4 At discontinuation 36

11 EFFICACY EVALUATION 37

11.1 NRS 37

11.2 EQ-5D-5L 37

12 SAFETY EVALUATION 37

12.1 Physical Examination 37

12.2 Laboratory Tests 38

12.3 Vital Signs 38

12.4 Biological Information Monitor 39

12.5 Pregnancy Test 39

12.6 Adverse Events 39

12.6.1 Definitions of adverse events 39

12.6.2 Severity of adverse events 40

12.6.3 Significant adverse events 40

12.6.4 Causal relationship with the investigational product 41

12.6.5 Observation of adverse events 41

12.6.6 Follow-up investigation and period after the onset of adverse events 41

12.7 Blood Drug Concentration Measurement 42

13 DISCONTINUATION CRITERIA AND PROCEDURES 42

13.1 Discontinuation Criteria 42

13.2 Discontinuation Procedures 43

14 SECURITY OF SAFETY OF SUBJECTS 44

14.1 Actions to Serious Adverse Events 44

14.1.1 Definition of serious adverse events 44

14.1.2 Handling of serious adverse events 44

14.2 Pregnancy 45

15 CLINICAL STUDY PROTOCOL COMPLIANCE, DEVIATION OR CHANGE, AND REVISION 45

15.1 Clinical Study Protocol Compliance 45

15.2 Clinical Study Protocol Deviations or Modifications 46

15.3 Clinical Study Protocol Amendment 46

16 COMPLETION, DISCONTINUATION, OR SUSPENSION OF THE STUDY 46

16.1 Completion of the Study 46

16.2 Discontinuation or Suspension of the Study 47

17 CASE REPORT FORMS 47

17.1 Format of the Case Report Form to Be Used in This Study 47

17.2 Entry to and Reporting with Case Report Forms 47

17.3 Review of Case Report Forms by the Investigator 48

17.4 Preparation and Reporting of the Patient Diary 48

18 SOURCE DOCUMENTS 49

18.1 Identification of Source Documents 49

18.2 Identification of Items for Which Data in Case Report Form Should Be Handled as Source Documents 49

18.3 Direct Access to Source Documents 49

19 STATISTICAL ANALYSIS 49

19.1 Analysis Populations 50

19.1.1 Subjects included in the efficacy analysis 50

19.1.2 Subjects included in the safety analysis 50

19.2 Statistical Analysis Items and Methods 50

19.2.1 Demographic and other baseline characteristics 50

19.2.2 Efficacy evaluation 50

19.2.3 Safety evaluation 52

19.2.4 Pharmacokinetic analysis (PK/PD and biomarkers) 52

19.2.5 Interval estimation and significance level 52

19.2.6 Handling of missing data, unscheduled observations, and other unusual data 52

19.2.7 Subgroup analyses 52

19.2.8 Exploratory analyses 53

19.2.9 Interim analysis 53

19.2.10 Additions and changes to the statistical analysis plan 53

20 QUALITY CONTROL AND ASSURANCE OF THE STUDY 53

20.1 Quality Control 53

20.2 Quality Assurance 54

21 ETHICAL CONDUCT OF THE STUDY 54

21.1 Compliance with GCP 54

21.2 Patient Information Sheet 54

21.2.1 Preparation of patient information sheet 54

21.2.2 Timing and method of obtaining informed consent 56

21.2.3 Revision of the patient information sheet 57

21.2.4 Other matters 57

21.3 Review by the IRB 57

21.4 Protection of Subjects' Human Rights 57

22 RETENTION OF RECORDS 58

22.1 Study Sites 58

22.2 The Founder of the Institutional Review Board 58

22.3 Sponsor 58

22.4 Notification from the Sponsor after the End of the Storage Period 59

23 PAYMENT AND INSURANCE 59

23.1 Compensation for Study-related Injuries and Insurance 59

23.2 Payment 59

24 PUBLICATION POLICY 59

25 STUDY ORGANIZATION 60

25.1 Sponsor 60

25.1.1 Sponsor 60

25.1.2 Clinical development manager 60

25.1.3 Clinical study manager 60

25.1.4 Medical expert 60

25.1.5 Coordinating investigator 61

25.1.6 Monitor 61

25.1.7 Data management manager 61

25.1.8 Statistical analysis manager 61

25.1.9 Quality control manager 62

25.1.10 Audit manager 62

25.1.11 Investigational product control manager 62

25.2 Contractor 62

25.2.1 Contract research organization (CRO) 62

25.2.2 Drug concentration measuring laboratories 64

25.3 Study Sites and Investigators 64

26 LIST OF SUPPORTING DATA 64

27 LIST OF ATTACHMENTS 64

28 REFERENCES 64

# HISTORY AND BACKGROUND OF DEVELOPMENT

## Overview of Investigational Products

NPC-06 is an injection containing fosphenytoin sodium hydrate as the active ingredient. The active ingredient is a preparation designed to eliminate adverse reactions to phenytoin at the injection site, such as pain and phlebitis. Because it is rapidly converted into the active substance, phenytoin, at the same molar equivalent (same molar quantity) in plasma, it can be intravenously administered without safety concerns. The active ingredient was approved for marketing as an intravenous preparation "Fostoin Intravenous Injection 750 mg" in July 2011 in Japan with the following indications: (1) status epilepticus, (2) suppression of epileptic seizures during cerebral surgery or consciousness disturbance (e.g., head trauma), and (3) temporary alternative therapy in patients with epilepsy receiving oral phenytoin.

### Physical, chemical, and pharmaceutical properties and formulation

The chemical name of fosphenytoin sodium hydrate is disodium(2,5-dioxo-4,4-diphenylimidazolidin-1-yl)methyl phosphate heptahydrate. Figure 1.1.1-1 shows the molecular formula, molecular weight, and structural formula of fosphenytoin sodium hydrate.

Structural formula:

Molecular formula: C_16_H_13_N_2_Na_2_O_6_P•7H_2_O

Molecular weight: 532.34 (406.24 as anhydride)

•7H_2_O

|  |  |
| --- | --- |

Figure 1.1.1-1 Structural Formula of Fosphenytoin Sodium Hydrate

NPC-06 contains 750 mg of fosphenytoin sodium per 10 mL in a vial. Store NPC-06 at 2°C to 8°C.

### Pharmacology

It is estimated that phenytoin binds to inactive Na^+^ channels to prolong the inactive state, thereby inhibiting the influx of Na^+^ and making nerve cell depolarization and axonal neurotransmission unlikely to occur. An *in situ* experiment on the effect of phenytoin on rabbit peripheral nerves showed that phenytoin increased the nerve stimulation threshold and eliminated nerve repeatability. Phenytoin prolonged the reaction latency of the trigeminal neural spinal canal to maxillary nerve stimuli in anesthetized cats.

In the mouse pain associated with acute herpes zoster model, the intravenous administration of 15 or 30 mg/kg of fosphenytoin significantly suppressed the induced pain response and spontaneous pain-like response.

### Pharmacokinetics in animals

The nonclinical pharmacokinetics of NPC-06 is summarized below.

- Comparable pharmacokinetic parameters of phenytoin were observed when fosphenytoin and phenytoin were intravenously administered in dogs.
- A high proportion of fosphenytoin (91% or higher) bound to dog and human plasma proteins (mainly albumin).
- It was inferred that fosphenytoin and its metabolites would remain in rat tissues only at low levels.
- Fosphenytoin was rapidly and nearly completely metabolized to phenytoin by alkaline phosphatase in rat and dog blood and tissues (in less than 1 minute in rats and up to 3 minutes in dogs).
- Comparable metabolites and excretion profiles were observed when fosphenytoin and phenytoin were intravenously administered in dogs.
- The major urinary metabolites in rats and dogs were p-HPPH glucuronide and m-HPPH glucuronide, respectively.
- Fosphenytoin was mainly excreted in urine and feces via biliary excretion in rats.

### Toxicity

Table 1.1.4-1 lists the toxicity study results of NPC-06.

Table 1.1.4-1 List of Toxicity Study Results

| Type of study | | Animal species  (Route of administration) | Results |
| --- | --- | --- | --- |
| Single-dose toxicity study | | Mouse  (continuous intravenous infusion) | LD_50_ 234 mg/kg |
|  |  | Rat (iv) | LD_50_ 319 mg/kg |
|  |  | Rat  (continuous intravenous infusion) | LD_50_ 363 mg/kg |
|  |  | Dog (iv) | No deaths occurred up to 60 mg/kg. |
|  |  | Dog  (continuous intravenous infusion) | No deaths occurred up to 60 mg/kg. |
| Repeated-dose toxicity study | | Rat (iv) | The NOAEL was 50 mg/kg/day when administered intravenously for 2 weeks. At 150 mg/kg/day, death, toxic signs, suppressed body weight gain, reduced food consumption, glucosuria, and increased urine output were observed.  The NOAEL was less than 30 mg/kg/day when administered intravenously for 4 weeks. Central nervous system symptoms were observed at 60 mg/kg/day or higher. Mild changes in the liver, such as increased glycogen accumulation, and local injection site injuries at the tail were observed in all dose groups. |
|  |  | Dog (iv) | The NOAEL was 15 mg/kg/day when administered intravenously for 2 weeks. Ataxia, salivation, decreased activity, vomiting, and gingival erythema were observed at 30 mg/kg/day or higher and soft stool/diarrhea, scleral hyperemia, and tremors were observed at 50 mg/kg/day.  The NOAEL was less than 15 mg/kg/day when administered intravenously for 4 weeks. Vomiting and mucous stools were sporadically observed at 15 mg/kg/day. Salivation, vomiting, mucous stools, erythema of the gums and nostrils, and ataxia were observed at 30 mg/kg/day or higher. Increased ALP was observed at 50 mg/kg/day. |
| Genotoxicity | Reverse mutation | *Salmonella typhimurium* | No mutagenicity was observed with or without metabolic activation. |
|  | Chromosomal aberration | CHL cells | No clastogenicity was observed with or without metabolic activation. |
|  | Micronucleus | Mouse (iv) | No clastogenicity was observed because there was no increase in micronucleated red blood cells. |
| Reproductive toxicity | Studies of fertility and early embryonic development until implantation (Seg 1) | Rat (im) | In a study in which treated males were mated with untreated females, toxicity was observed in males at 75 mg/kg or higher, but no abnormalities were observed in female fertility and early embryonic development.  In a study in which treated females were mated with untreated males, toxicity and reproductive disorders were observed in females at 75 mg/kg or higher, resulting in growth suppression of F_1_ animals. At 25 mg/kg or higher, fetal developmental disorders including teratogenicity were observed. |
|  | Studies of embryo-fetal development (Seg 2) | Rat (iv) | Dams at 100 mg/kg showed reduced body weight and food consumption: 4 dams died. In fetuses, decreased body weight and increasing tendency of bone abnormalities were observed at 100 mg/kg. In F_1_ animals, reduced number of offspring and suppressed body weight gain (13 weeks of age) in F_1_ males were observed at 100 mg/kg. |
|  |  | Rabbit (iv) | Although no dams died, body weight and food consumption were reduced at 25 mg/kg or higher. However, there was no fetal toxicity or teratogenicity. |
|  | Studies of effects on prenatal and postnatal development and maternal function (Seg 3) | Rat (iv) | Maternal body weight and food consumption were reduced at 100 mg/kg and the gestation period was prolonged at 50 mg/kg or higher.  In neonates, the following changes were observed: reduced survival rate and body weight at 100 mg/kg; increased structural variations including dilatation of the ureter or renal pelvis and reduced renal papilla at 50 mg/kg or higher; and mild increase in activity and increase in avoidance behavior. However, there was no effect on the fertility or reproductive function of F_1_ animals. |
| Local tolerance (comparison with phenytoin) | Vascular and perivascular tissues | Rabbit  (infusion, sc) | Fosphenytoin (up to 75 mg/mL) irritated vascular and perivascular tissues to the same degree as physiological saline (control). Phenytoin showed higher irritating effects than the control at 16.9 mg/mL or higher and increased thrombus formation at 33.7 mg/mL or higher. Fosphenytoin was less irritating to vascular and perivascular tissues than phenytoin. |

### Clinical pharmacokinetics

NPC-06 is rapidly converted to phenytoin in the body by alkaline phosphatase or acid phosphatase in the blood. One mmol of fosphenytoin is converted to 1 mmol of phenytoin. When converted to phenytoin, phosphate and formaldehyde are produced and formaldehyde is converted to formate and metabolized.

(1) Pharmacokinetics of plasma fosphenytoin

| Absorption/bioavailability | When NPC-06 is intravenously administered, the plasma fosphenytoin concentration is maximized at the end of administration. The half-life of fosphenytoin is approximately 15 minutes. |
| --- | --- |
| Distribution | A high proportion of fosphenytoin (95% to 99%) binds to human plasma proteins, mainly albumin. Because the binding rate decreases with the increase in total fosphenytoin concentration, the binding to plasma proteins is saturated. Fosphenytoin is replaced by phenytoin at the protein binding site. The volume of distribution of fosphenytoin ranges from 4.3 to 10.8 L and increases with the dose and infusion rate of NPC-06. |
| Metabolism and excretion | The half-life of fosphenytoin by the conversion to phenytoin is approximately 15 minutes. Although the conversion mechanism of fosphenytoin is not clear, it seems that phosphatase plays an important role. Fosphenytoin is not excreted in urine. One mmol of fosphenytoin is metabolized to 1 mmol of phenytoin, phosphate, and formate. |

(2) Pharmacokinetics of plasma phenytoin

| Absorption/bioavailability | NPC-06 is fully converted to phenytoin after intravenous administration and its half-life is approximately 15 minutes. |
| --- | --- |
| Distribution | A high proportion of phenytoin binds to plasma proteins, mainly albumin, although the proportion is less than that of fosphenytoin. In the absence of fosphenytoin, approximately 12% of the plasma total phenytoin remains unbound in the concentration range of the clinical dose. However, fosphenytoin is replaced by phenytoin at the binding site to plasma proteins. Therefore, the proportion of free phenytoin is increased (up to 30%) while fosphenytoin is converted to phenytoin (approximately 0.5 to 1 hour after administration). |
| Metabolism and excretion | The phenytoin produced by NPC-06 is metabolized in the liver. NPC-06 is excreted in urine mainly as 5- (p-hydroxyphenyl)-5-phenylhydantoin and its glucuronide conjugate, and almost no unchanged drug is detected in urine (1% to 5% of the dose). The hepatic metabolism of phenytoin is saturated. After a single intravenous administration of 600 to 1,800 mg, the AUC of total and free phenytoin increased more than dose proportionally. The mean half-life of the total phenytoin level following administration of NPC-06 at this dose level (12.0 to 28.9 h) is similar to that following the parenteral administration of the equivalent dose of phenytoin and tends to be longer at higher plasma phenytoin levels. |

(3) Drug interactions

There is no drug known to inhibit the conversion of fosphenytoin to phenytoin. Therefore, we estimate that the drug interactions after administration of NPC-06 are the same as those known for phenytoin. Phenytoin is metabolized mainly by CYP2C9 in the liver and partially by CYP2C19 ^1,2)^. Phenytoin is known to have hepatic metabolic enzyme-inducing activity. Particularly, it induces CYP3A4, CYP2B6, and P-glycoproteins ^3)^.

### Clinical efficacy

No study has reported the efficacy of NPC-06 for pain associated with acute herpes zoster. However, several case reports are available for the efficacy of phenytoin for postherpetic neuralgia. ^4,5)^

### Clinical safety

The Japanese clinical studies performed for the marketing approval of NPC-06 in July 2011 included the Phase I study in healthy adults and the Phase III study in patients with status epilepticus or repetitive status epilepticus or patients after brain surgery. Adverse events observed at a high frequency (≥ 5%) after the administration of NPC-06 included pyrexia, dizziness, headache, nausea, blood pressure decreased, vomiting, feeling abnormal, nystagmus, somnolence, erythema, hepatic function abnormal, pruritus, and changes in laboratory test parameters including white blood cell count increased, C-reactive protein increased, blood glucose increased, blood albumin decreased, total protein decreased, haemoglobin decreased, red blood cell count decreased, haematocrit decreased, and differential white blood cell count abnormal. Adverse reactions observed at a high frequency (≥ 5%) included dizziness, blood pressure decreased, feeling abnormal, nystagmus, headache, pruritus, and pyrexia. No serious adverse events were observed in the Japanese clinical studies.

In non-Japanese clinical studies in which NPC-06 was administered in a total of 988 subjects, serious adverse events possibly related to NPC-06 occurred in 8 subjects (0.8%), including temporary psychotic disorder after seizure in 2 subjects and hypotension/cardiac arrest/stupor, hypotension/bradycardia, overdose (phenytoin injection), overdose (carbamazepine), nystagmus/ataxia, and coma in 1 subject each.

## History of Development

Varicella zoster virus (VZV) develops varicella at the primary infection. Although varicella heals spontaneously, VZV remains latent in ganglions after varicella heals and is reactivated after years by aging, stress, overwork, or use of an immunosuppressant. The recurrent infection with VZV transmits to the skin through the nerves, while causing nerve inflammation and damage. It then results in the development of herpes zoster associated with characteristic rash and pain. Although the rash generally heals in 28 days, it is associated with prolonged inflammation causing irreversible nerve damage, resulting in the onset of postherpetic neuralgia with hyperalgesia and allodynia in some patients.

Pain associated with acute herpes zoster is mainly nociceptive pain, while postherpetic neuralgia is neuropathic pain. The mechanism of pain differs between the two. Although pain associated with acute herpes zoster and postherpetic neuralgia were considered separately, they are recently regarded as continuous pain and called "zoster-associated pain (ZAP)" ^6,7)^.

Herpes zoster is generally treated with antiviral agents, and analgesics such as NSAIDs and acetaminophen (hereinafter referred to as non-opioid analgesics) are prescribed for pain as the standard of care. In some cases, drugs for neuropathic pain are prescribed as an analgesic adjuvant because non-opioid analgesics alone fail to manage severe pain in the acute phase.

With the main activity to inhibit the Na^+^ channel ^8)^, phenytoin inhibits the influx of Na^+^ to make nerve cell depolarization and axonal neurotransmission unlikely to occur ^9,10)^. By this mechanism, phenytoin is believed to specifically inhibit only abnormally high frequency of nerve ‘firing’ and does not suppress normal nerve activity with low firing frequency, and therefore is expected to be useful in alleviating neuropathic pain.

Although there has been no report of fosphenytoin administered in patients with pain associated with acute herpes zoster, Hatangdi ^4)^ examined the effect of carbamazepine or phenytoin in combination with antidepressants on postherpetic neuralgia and reported satisfactory results in patients receiving phenytoin. Braham et al. ^5)^ reported that the oral administration of phenytoin eliminated repeated sudden severe pain attacks in 2 patients with postherpetic neuralgia on the chest.

Recently, NPC-06 was administered in an acute herpes zoster mouse model to examine its pharmacological properties for pain associated with acute herpes zoster. Alopecic mice that were inoculated with HSV-1 (herpes simplex virus) and showed herpes zoster-like rash and stable pain-like behaviors were included to measure induced pain and spontaneous pain-like behaviors on Day 6 after the inoculation with HSV-1. It was confirmed that NPC-06 suppressed induced pain (isomeric pain and hyperalgesia) and spontaneous pain-like behaviors in the mouse model and, based on the results, it was expected to relieve pain associated with acute herpes zoster. Therefore, the sponsor planned this study (Phase II study) to clinically investigate the analgesic effect of NPC-06 on acute pain in patients with pain associated with acute herpes zoster. In addition, the Pharmaceuticals and Medical Devices Agency was consulted to make this plan.

# OBJECTIVE

## Objective of the Study

This study is intended to examine the pain-relieving effect and safety of NPC-06 (fosphenytoin sodium injection) in patients with pain associated with acute herpes zoster in a placebo-controlled, double-blind, parallel-group, comparative manner. This study is also intended to explore the effective concentration range of the test drug.

## Phase of Development

Phase II exploratory study

# STUDY DESIGN

## Study Design

Multicenter, randomized, placebo-controlled, double-blind, parallel-group, comparative study

## Group Structure

1. Test drug group (NPC-06 group): Fosphenytoin sodium (750 mg per 10 mL in a vial)
2. Control group (placebo group): 10 mL of isotonic sodium chloride solution in a vial

## Dosage and Administration Method

Subjects will be randomly assigned to any of the following 4 dose groups: NPC-06 high-dose and placebo groups as the high-dose groups and NPC-06 low-dose and placebo groups as the low-dose groups.

The high-dose group will receive an intravenous drip infusion of 18 mg/kg of NPC-06 or placebo over at least 18 minutes per dose.

The low-dose group will receive an intravenous drip infusion of 12 mg/kg of NPC-06 or placebo over at least 12 minutes per dose.

In this regard, however, the dosage of NPC-06 must not exceed 1,200 mg on the basis of fosphenytoin sodium in both dose groups.

For convenience, subjects treated with NPC-06 in the high-dose and low-dose groups are hereinafter referred to as the high-dose group and the low-dose group, respectively. Subjects treated with placebo are referred to as the placebo group.

<Rationale for the dosage>

NPC-06 is generally considered effective for status epilepticus when the blood concentration of total phenytoin is 10 to 20 μg/mL. NPC-06 has been reported to be used at 11 to 18 mg/kgPE (equivalent to 16.5 to 27 mg/kg of fosphenytoin) for the treatment of neuropathic pain in foreign countries. In Japan, a clinical experience of using NPC-06 in patients with trigeminal neuralgia has been reported in which a single dose of 750 mg/patient (15 mg/kg assuming a body weight of 50 kg) relieved pain. Based on these results, NPC-06 is expected to be effective for pain in the dose range used for status epilepticus or for inhibiting seizures after brain surgery (15 to 22.5 mg/kg). In this study, the following working hypothesis is made: NPC-06 is more effective than placebo and provides the effect for pain at the same level as the lower limit of the effective concentration for epilepsy (10 μg/mL). At 18 mg/kg, it is estimated that the total phenytoin level in the blood exceeds 10 μg/mL for 12 to 13 hours. At 12 mg/kg, it is estimated that the total phenytoin level in the blood always falls below 10 μg/mL except at the peak. It is therefore decided that a high-dose group (18 mg/kg) and low-dose group (12 mg/kg) will be set as parallel groups, and that the results from the 2 groups will be used to determine the effective concentration range for pain.

In addition, the maximum limit of the dosage in the NPC-06 groups is set at 1,200 mg because the clinical study in non-Japanese healthy adults showed that nervous system disorders, such as nystagmus, dizziness, and paraesthesia, occurred as adverse events at a markedly increased incidence in those treated with more than 1,200 mg as compared to those treated with 1,200 mg or lower of NPC-06.

<Rationale for the infusion rate>

Because cardiac rhythm disorders, such as bradycardia and cardiac arrest, were reported when NPC-06 was rapidly administered intravenously, it was specified that the infusion rate should not exceed 1 mg/kg/min (approved infusion rate range), with reference to the approved infusion rate range.

<Rationale for the comparator>

To objectively evaluate the efficacy of NPC-06, it was specified that the test drug should be compared with the placebo preparation containing 10 mL of isotonic sodium chloride solution in the same vial in a blinded manner.

<Rationale for the duration of treatment>

The primary objective of this study is to obtain evidence of the rapid pain relief effect of the test drug for pain associated with acute herpes zoster. The previous study on the systemic administration of lidocaine in patients with pain associated with acute herpes zoster ^11)^ reported that none of the patients treated with the drug 1 to 3 times a week required nerve block therapy. Based on this report, the single-dose administration of NPC-06 does not cover the whole acute pain period, but is considered valid as the treatment for relieving at least the severest pain. The primary objective of the study is achieved if pain relief by a single dose is demonstrated.

## Target Number of Subjects

48 subjects

16 subjects in the NPC-06 high-dose group and 8 subjects in the placebo group

16 subjects in the NPC-06 low-dose group and 8 subjects in the placebo group

< Rationale for target sample size >

Because the effective dose of NPC-06 to relieve pain associated with acute herpes zoster is not known, the target number of subjects is set as the feasible and minimum number of subjects required for evaluating efficacy.

Given the study design, it is considered appropriate to compare each dose group of NPC-06 (high- and low-dose groups) with the placebo group. Because an allocation ratio of 1 to 1 between the groups is considered to provide the highest statistical power, the number of subjects in each dose group of NPC-06 and placebo group was set at 16. In the placebo group, 8 subjects each will receive the placebo in the same manner as in the high-dose and low-dose groups, respectively. A total of 16 subjects in the placebo group will be compared with each dose group during the analytical process. The analytical process will proceed according to the closed testing procedure in the order of [1] high-dose group vs. placebo group followed by [2] low-dose group vs. placebo group, considering multiplicity. The population consisting of 16 subjects in the NPC-06 group and 16 subjects in the placebo group had a power of 86% when α was 5% (2-sided) for the mean reduction in the NRS score by 2 points in the NPC-06 group as compared to the placebo group (assuming SD of 1.8) ^Note)^.

Note) Fujii et al. determined an effect size (Cohen’s d) of 1.11 based on the pain relief score (PRS) for acute pain in herpes zoster using lidocaine and physiological saline ^11)^. A comparable effect size was expected for NPC-06 because NPC-06 and lidocaine have the same mechanism of action. It was then applied to the NRS score and the SD was determined to be 1.8 when the difference between NPC-06 and placebo was 2.

## Study Period

June 2019 to March 2021

# PATIENT POPULATION

## Target Disease

Pain associated with acute herpes zoster

## Inclusion Criteria

Patients with pain associated with acute herpes zoster who meet all the following criteria (1) to (9) at the subject registration will be included.

1. Patients aged 20 years or older at the acquisition of informed consent
2. Sex: Either male or female
3. Patients with herpes zoster who present with acute onset of skin rash (single or concurrent occurrence of erythema, papule, blister, and pustule). However, patients who have crust formation of more than 20% of the whole rash immediately before the start of the study treatment will be excluded.
4. Patients who are on an antiviral drug for herpes zoster at the start of the study treatment
5. Patients who are on a non-opioid analgesic (acetaminophen or NSAIDs) for herpes zoster at the start of the study treatment.
6. Patients with pain intensity of ≥ 4 NRS score at the following evaluation points

- 120 minutes before the start of the study treatment

- Immediately before the start of the study treatment

1. Patients with poor response to non-opioid analgesics for herpes zoster

The term “poor response” is defined as a condition that meets both of the following criteria.

- The mean pain intensity is ≥ 4 NRS score in spite of the treatment with non-opioid analgesics from 24 hours to 120 minutes before the start of the study treatment (non-opioid analgesics are used at least twice during the period).

- The reduction in NRS score at immediately before the start of the study treatment (120 minutes after the administration of non-opioid analgesics) is less than 2 points in comparison with the score at 120 minutes before the start of the study treatment (before the administration of non-opioid analgesics) despite the treatment with non-opioid analgesics before the study treatment.

1. Patients who can be hospitalized
2. Patients for whom written informed consent was obtained with the patient’s own or legally acceptable representative’s free will with full understanding after receiving sufficient explanation. In the case where a legally acceptable representative is required for the patient, written informed consent must be obtained from both the patient and his or her legally acceptable representative.

<Rationale>

(1) Herpes zoster is more likely to occur in patients aged 20 years or older.

(2) No effect of sex on efficacy is known.

(3) This criterion is intended to evaluate the effect on pain associated with acute herpes zoster. Because acute pain is likely to be relieved before rash gets crusted, the range of crust formation immediately before the start of the study treatment is specified.

(4) and (5) These criteria were set to select patients receiving treatment for acute herpes zoster.

(6) This criterion was set because an NRS score of at least 4 was required to appropriately evaluate pain. Additionally, an NRS score of at least 4 was set with reference to the design of the studies of lidocaine for central neuropathic pain ^12)^ and opioid-refractory cancer pain ^13)^.

(7) NPC-06 is intended for patients who need quick pain relief among those who have pain associated with acute herpes zoster and poor response to non-opioid analgesics used as the standard of care, resulting in difficult pain management, or those who need the treatment with analgesic adjuvants. Patients with poorly controlled pain are defined as those with the mean pain intensity of ≥ 4 NRS score in spite of the treatment with antiviral agents or non-opioid analgesics.

Patients are considered to have poor response to non-opioid analgesics when the NRS score at 120 minutes after administration is increased, not changed, or reduced by less than 2 points. In the pivotal efficacy study of pregabalin for postherpetic neuralgia (Study 1008-169), a reduction in the NRS score by 2 or more was considered to indicate clinical significance, although the study was performed in patients with a different type of pain.

(8) NPC-06 is intended to be used regardless of hospitalization status after marketing. Hospitalization management is considered necessary for this study to appropriately evaluate the efficacy of the investigational products and ensure the safety of subjects. Subjects will include patients who will remain hospitalized after the completion of the study.

(9) This criterion was set to perform the study in compliance with GCP.

## Exclusion Criteria

Patients who meet any of the following criteria at the subject registration will be excluded.

1. Patients who cannot evaluate pain with NRS
2. Patients suspected to have increased intracranial pressure
3. Patients complicated with epilepsy, serious neuropsychiatric disorders (e.g., dementia, Parkinson's disease, or schizophrenia), or consciousness disturbance
4. Patients complicated with malignant tumors (excluding patients who are under treatment management and show no sign of recurrence), patients being treated for HIV, and patients being treated with immunosuppressants (including biologics) (excluding patients who have no hindrance in daily life and whose general condition is favorable)
5. Patients complicated with idiopathic trigeminal neuralgia
6. Patients with other severe pain that may affect the assessment of pain associated with acute herpes zoster
7. Patients being treated with opioid analgesics or steroid anti-inflammatory drugs (systemic) for analgesia of pain associated with acute herpes zoster
8. Patients with sinus bradycardia or advanced conduction disturbance
9. Patients with a history of hypersensitivity to hydantoin drugs
10. Patients being treated with tadalafil (for pulmonary hypertension), rilpivirine hydrochloride, asunaprevir, daclatasvir hydrochloride, macitentan, elbasvir, grazoprevir hydrate, ticagrelor, artemether・lumefantrine, daclatasvir hydrochloride・asunaprevir・beclabuvir hydrochloride, darunavir ethanolate・cobicistat, rilpivirine hydrochloride・tenofovir disoproxil fumarate・emtricitabine, rilpivirine hydrochloride・tenofovir alafenamide fumarate・emtricitabine, bictegravir sodium・emtricitabine・tenofovir alafenamide fumarate, elvitegravir・cobicistat・emtricitabine・tenofovir alafenamide fumarate, elvitegravir・cobicistat・emtricitabine・tenofovir disoproxil fumarate, sofosbuvir・velpatasvir, sofosbuvir, ledipasvir acetonate・sofosbuvi, or dolutegravir sodium・rilpivirine hydrochloride
11. Patients being treated with drugs for neuropathic pain, antidepressants, antiarrhythmics, NMDA receptor antagonists, central muscle relaxants, local anesthetics, vaccinia virus inoculated rabbit inflamed skin extract, Chinese herbal medicines and vitamin B_12_ preparations for analgesia of the pain associated with acute herpes zoster
12. Patients treated with amenamevir within 24 hours before the start of the study treatment
13. Patients complicated with meningitis or patients with meningeal irritation symptoms
14. Patients with a serious cardiac disease, respiratory disorder, or hepatic/renal impairment (the term “serious” refers to Grade 3 in the Standards for Classification of Seriousness of Adverse Drug Reactions by Drugs etc. (PAB/SD Notification No. 80))
15. Patients being treated with fosphenytoin, phenytoin, ethotoin, or any combination drug containing any of these drugs, or patients who received any of these drugs as an analgesic adjuvant
16. Patients who participated in another clinical trial within 3 months before the date of the screening test
17. Pregnant or lactating women or patients who may become pregnant during the study period
18. Patients unable to agree to use appropriate contraception as instructed by the investigator or subinvestigator (hereinafter referred to as the investigators) during the period from the acquisition of informed consent to the end of the post-treatment observation period
19. Other patients inappropriate for the participation in this study, as judged by the investigators

<Rationale>

(1) The severity of pain used as an endpoint is based on the patient's self-assessment.

(2) to (7) These diseases may affect efficacy evaluation.

(8) to (10) These diseases/drugs are included in the contraindications specified in the approved package insert of the test drug.

(11) These diseases may affect efficacy evaluation.

(12) CYP3A4 may be induced and the effect of amenamevir may be reduced.

(13) and (14) These criteria were set to secure patient safety.

(15) The selection bias of subjects may be involved.

(16) to (19) These criteria are included as general considerations for the study.

# REGISTRATION OF SUBJECTS

The investigators will register subjects who meet the enrollment criteria (inclusion and exclusion criteria). Registered subjects will be assigned to the test drug (NPC-06) or control drug (placebo) according to 5.2 Registration Procedure.

## Preparation of Subject Screening and Subject Registration Lists

The investigators will prepare a subject screening list including the subject identification code to protect privacy, the subject name, the medical chart number, and the date of obtaining informed consent. The subject screening list will include all candidate subjects who received the explanation of the study to obtain consent before starting the study.

## Registration Procedure

Subjects will be registered according to the following procedure.

1. The investigators will obtain voluntary written consent from each candidate subject who may meet the enrollment criteria of the study.
2. The investigators will then enter the necessary items, such as the conformance to the enrollment criteria, to the Electronic Data Capture (EDC) for subjects who provided the consent to participate in the study.
3. A randomized drug number will be automatically assigned to each subject whose information entered in the EDC meets the enrollment criteria.

## Preparation of Investigational Product Assignment Table, Blinding Method, and Maintenance of Blinding

### Storage of investigational product assignment table and emergency code

This study will be conducted in a placebo-controlled, randomized, double-blind, parallel group design to compare the test drug with placebo. The permuted block method will be used for randomization, with a block size of each dose group of 3 and ratio of the test drug to placebo of 2 to 1. The assignment of the investigational products will be blinded to the subjects, investigators, and all clinical research coordinators/those involved in the study. The investigational products will be kept blinded by making the appearance and packaging form of the test drug and placebo indistinguishable.

The investigational products will be blinded through the study period by the following procedures.

- The investigational product assignment manager will prepare an investigational product assignment table.
- The investigational product assignment manager will guarantee the indistinguishability of the appearance, packaging form, and labeling of the investigational products.
- The investigational product assignment manager will enclose the assignment table in an envelope, seal the envelope, and keep it safe under lock and key until the blind is broken.
- Unused investigational products or remaining drugs to be collected before unblinding, if any, should be collected in a sealed state by the investigational product administrator to maintain the blind.

The investigational product assignment manager will prepare 2 sets of emergency codes (original and duplicate) so that individual subjects can be unblinded in case of emergency, such as the onset of a serious adverse event. The investigational product assignment manager and sponsor will keep one set each of the emergency codes and will not open them except when they need to be opened through the specified procedure (see 5.3.3 Unblinding procedure during the study).

### Unblinding procedure

The investigational product assignment manager will break the code after the data from all subjects are entered into the EDC, the SDV is completed, handling of data for analytical purposes is decided, and data are fixed.

### Unblinding procedure during the study

If the investigators judge it necessary to know the drug assignment to secure subject safety when, for example, a serious adverse event has occurred, the investigators will promptly enter the details about the subject in question in the Serious Adverse Event Report and submit it to the sponsor. In that case, the investigators can request the sponsor to open the emergency code of the subject. If the sponsor judges it necessary to open the emergency code based on the information reported, the investigational product assignment manager will open the original emergency code or the sponsor will open the duplicate copy of the emergency code and promptly inform the investigators of the subject’s drug assignment. The sponsor will document the date and reason for unblinding. The sponsor will make efforts to ensure that the drug assignment will be disclosed only to the investigators who requested unblinding, subject in question, and clinical research coordinator(s) responsible for the subject. If the drug(s) in the same block (set) as the unblinded drug remain unassigned, it must not be used because, if used, it may increase the predictability of assignment.

The subject for whom the emergency code is opened will be withdrawn from the study at the time of opening. The data collected from the subject before withdrawal will be handled in the same manner as those from the other patients for whom assignment is still blinded.

# INVESTIGATIONAL PRODUCTS

## Name and Other Information of Investigational Products

1. Test drug

Test drug name: NPC-06

Nonproprietary name: Fosphenytoin Sodium Hydrate (JAN)

Chemical name: Disodium(2,5-dioxo-4,4-diphenylimidazolidin-1-yl)methyl phosphate heptahydrate

Molecular formula: C_16_H_13_N_2_Na_2_O_6_P•7H_2_O

Molecular weight: 532.34

1. Control drug

Control drug name: Japanese Pharmacopoeia (JP) Isotonic Sodium Chloride Solution

Nonproprietary name: Sodium chloride

Chemical name: Sodium Chloride

Molecular formula: NaCl

Molecular weight: 58.44

## Components/content and Dosage Form

1. Test drug

NPC-06: Colorless to pale yellow, clear solution for injection containing 750 mg of fosphenytoin sodium per 10 mL in a vial

1. Control drug

Placebo: Ten mL per vial of JP Isotonic Sodium Chloride Solution (colorless and clear solution for injection indistinguishable from the test drug by appearance)

### Packaging form

Two vials of the investigational product will be put in an individual package, which will be sealed for 1 subject. Inner boxes for 6 subjects will be put in an outer box, which will be used for 1 set.

### Labeling

The following items are labeled on the inner and outer boxes.

- The contents of the box are for investigational use.
- Number of assignment set (outer box) or assignment set number (inner box)
- Name and address of the sponsor
- Identification code
- Clinical study protocol number
- Manufacturing number
- Storage method
- Quantity

## Control of Investigational Products

The investigational products will be controlled according to the Investigational Product Handling Procedure.

### Storage

Store in a cool place (2°C to 8°C).

Store according to the Investigational Product Handling Procedure.

### Shelf life

The shelf life of the investigational products is separately described in the Investigational Product Handling Procedure.

### Delivery

Upon the conclusion of the agreement with study sites, the sponsor will deliver the investigational products to the study sites.

### Storage and control

The investigational product administrator will properly store and control the investigational products and record the use of the investigational products in compliance with the following procedure. The investigational products are intended only for this clinical study and may not be used for other purposes.

1. The sponsor will issue the Investigational Product Handling Procedure to study sites at or before the delivery of the investigational products to the sites.
2. The investigational product administrator will store and control the investigational products according to the Investigational Product Handling Procedure and prepare the investigational product control table to record the prescription and use by subjects at each study site.
3. After the preparation of the investigational products, the investigators, investigational product administrator, and clinical research coordinators will keep the drug remaining in each vial, if any, until the collection of the investigational products. The investigational products remaining in a drip infusion bag for reasons such as administration discontinuation, if any, should be discarded according to the procedure specified at each study site.
4. Upon completion of the study, the investigational product administrator will promptly return unused investigational products, used vials, and packaging boxes to the sponsor together with the investigational product control table (copy) (see 6.3.5 Collection).

### Collection

The sponsor will collect unused investigational products, used vials, and packaging boxes from the investigational product administrator. The sponsor will receive a copy of the investigational product control table.

When any investigational product is returned to the sponsor before unblinding, the investigational product administrator will check the quantity of the remaining investigational products, seal the box(es) of the investigational products, and return them to the sponsor.

# ADMINISTRATION OF THE INVESTIGATIONAL PRODUCTS AND MONITORING OF SUBJECTS

## Dosage and Administration Method of the Investigational Products

This is a single-dose study (Day 1) and the dosage and administration of the investigational product in each group is as follows.

[High-dose group]

<Dose>

Eighteen mg/kg of NPC-06 or placebo will be administered once by intravenous drip infusion.

<Administration method>

The investigational product will be diluted 3 to 4-fold with a physiological saline solution for drip infusion and infused over 18 minutes or longer.

[Low-dose group]

<Dose>

Twelve mg/kg of NPC-06 or placebo will be administered once by intravenous drip infusion.

<Administration method>

The investigational product will be diluted 3 to 4-fold with a physiological saline solution for drip infusion and infused over 12 minutes or longer.

In this regard, however, the dosage of NPC-06 must not exceed 1,200 mg on the basis of fosphenytoin sodium in both dose groups.

<Rationale for dosage, infusion rate, and duration of administration>

See Section 3.3.

## Preparation of Investigational Products

One vial (10 mL) of the test drug contains 750 mg (75 mg/mL) of fosphenytoin sodium. One vial of the control drug (placebo) contains 10 mL of isotonic sodium chloride solution. The necessary volume (mL; rounded to the nearest whole number) of the investigational product will be determined based on the body weight of each subject. It will be diluted 3 to 4-fold with physiological saline for injection. The infusion bag containing the investigational product will be set on the infusion stand or pump. The investigational product remaining in each opened vial after preparation must not be used.

<Example of preparation of investigational product>

The necessary volume of the investigational product is shown below using an example based on a subject weighing 50 kg.

[High-dose group]

1. Dose: 18 mg/kg × 50 kg = 900 mg
2. Necessary volume of investigational product: 900 mg / 75 mg/mL = 12 mL (rounded off to the nearest whole number, if applicable)

[Low-dose group]

1. Dose: 12 mg/kg × 50 kg = 600 mg
2. Necessary volume of investigational product: 600 mg / 75 mg/mL = 8 mL (rounded off to the nearest whole number, if applicable)

<Precautions>

Fully fill the infusion line up to the tip with the investigational product solution before starting drip infusion. Flush the infusion line with physiological saline to remove any remaining investigational product.

## Monitoring of Subjects

At administration, blood pressure, heart rate, respiratory rate, ECG, and SpO_2_ will be monitored using the biological information monitor from immediately before to 120 minutes after the start of the study treatment. The infusion of the investigational product will be immediately discontinued and necessary treatment given if reduced blood pressure meeting the discontinuation criteria or clinically significant abnormalities such as cardiac depression and respiratory disorder occur. Abnormal findings and treatments given for them will be recorded in source documents and entered into the EDC.

# CONCOMITANT DRUGS, CONCOMITANT THERAPIES, AND OTHER RESTRICTIONS

## Prohibited Concomitant Drugs

The following drugs are prohibited for the respective specified periods. Subjects who require any of these drugs after the start of the study treatment will be withdrawn from the study.

Prohibited pretreatment or concomitant drugs from 2 weeks before the start of the study treatment to the end of the evaluation at 24 hours after administration on the next day of the study treatment (Day 2)

- Phenytoin, fosphenytoin, ethotoin, and combination drugs containing any of these drugs
- Antiepileptic drugs, tadalafil, rilpivirine hydrochloride, asunaprevir, daclatasvir hydrochloride, macitentan, elbasvir, grazoprevir hydrate, ticagrelor, artemether・lumefantrine, daclatasvir hydrochloride・asunaprevir・beclabuvir hydrochloride, darunavir ethanolate・cobicistat, rilpivirine hydrochloride・tenofovir disoproxil fumarate・emtricitabine, rilpivirine hydrochloride・tenofovir alafenamide fumarate・emtricitabine, bictegravir sodium・emtricitabine・tenofovir alafenamide fumarate, elvitegravir・cobicistat・emtricitabine・tenofovir alafenamide fumarate, elvitegravir・cobicistat・emtricitabine・tenofovir disoproxil fumarate, sofosbuvir・velpatasvir, sofosbuvir, ledipasvir acetonate・sofosbuvi, or dolutegravir sodium・rilpivirine hydrochloride
- Drugs for neuropathic pain, antidepressants, antiarrhythmics, NMDA receptor antagonists, central muscle relaxants, and local anesthetics
- Opioid analgesics
- Steroidal anti-inflammatory drugs (systemic) for analgesia of pain associated with acute herpes zoster
- Vaccinia virus inoculated rabbit inflamed skin extract
- Chinese herbal medicines for analgesia of pain associated with acute herpes zoster
- Vitamin B_12_ preparations for analgesia of pain associated with acute herpes zoster

Prohibited pretreatment or concomitant drugs from 24 hours before the start of the study treatment to the end of the evaluation at 24 hours after administration on the next day of the study treatment (Day 2)

- Amenamevir
- Sustained release non-opioid analgesics

## Prohibited Concomitant Therapies

The following therapies are prohibited for the respective specified periods. Subjects who require any of these therapies after the start of the study treatment will be withdrawn from the study.

Prohibited pretreatment or concomitant therapies from the onset of herpes zoster to the end of the evaluation at 24 hours after administration on the next day of the study treatment (Day 2)

- Nerve block therapy for the efficacy evaluation site
- Anti-inflammatory analgesic treatment (e.g., low-power laser therapy, low-power ultrasonic therapy)
- Other surgical treatments for pain

## Restricted Concomitant Drugs

The switch to another drug, change in the route of administration or dosage and administration of the current drugs, or addition of a new drug is prohibited for the following drugs from the onset of herpes zoster to the end of the evaluation at 24 hours after administration on the next day of the study treatment (Day 2)

- Aspirin preparations for antiplatelet action
- Steroidal anti-inflammatory drugs (systemic)
- Vitamin B_12_ preparations
- Immunosuppressants (including biologics)

## Other Restrictions

The investigators will explain the following contraceptive methods to subjects and instruct them to take appropriate contraceptive measures with their partners during the period from the acquisition of informed consent to the end of the post-treatment observation period.

Contraceptive methods for subjects and their partners:

- Intrauterine contraceptive device
- Intrauterine contraceptive system
- Oral contraceptives
- Contraceptive surgery
- Double-barrier method (e.g., condoms with spermicide and combination of condom and diaphragm)
- Abstinence

# ENDPOINTS

## Primary Endpoint

Slope in the NRS score over the evaluation period (mean score change) based on the NRS score before (baseline) and 30, 60, 90, and 120 minutes after the start of the study treatment

<Rationale>

NRS is one of the most commonly used pain rating scales. The primary endpoint was set to evaluate the rapid analgesic effect of NPC-06 using the NRS score, which is a reliable and validated evaluation scale. The NRS score will be recorded immediately before (baseline) and 30, 60, 90, and 120 minutes after the start of the study treatment to analyze the mean score change over the period from the baseline to 120 minutes after the start of the study treatment. Fujii, et al ^9)^ administered lidocaine, which was confirmed effective for neuropathic pain, to pain associated with acute herpes zoster systemically (intravenous drip infusion) over 30 minutes. Lidocaine was found effective based on the pain evaluation immediately after administration. Therefore, it was specified that the NRS score should be evaluated for 120 minutes, including the drip infusion of NPC-06, to cover the period of about 60 minutes before and after administration.

Additionally, pain associated with acute herpes zoster is resolved spontaneously due to the effect of the antiviral agent. Therefore, the prolonged evaluation period may affect the evaluation of pain intensity. The period of 120 minutes after the start of the study treatment was set to minimize the effect and consider the time to develop the pain relief effect and therefore considered appropriate.

## Secondary Endpoints

- 1. Change in the NRS score at each evaluation point from the baseline
  2. Proportion of subjects in whom the NRS score is improved by at least 2 points at 120 minutes after the start of the study treatment as compared to the baseline
  3. Change in QOL value of EQ-5D-5L at each evaluation point from the baseline

<Rationale>

(1) This criterion was set to evaluate the change in the analgesic effect from 4 to 24 hours after the start of the study treatment using the NRS score to examine the efficacy of NPC-06.

(2) In the pivotal efficacy study of pregabalin for postherpetic neuralgia (Study 1008-169), the change in the pain score (primary endpoint; pain in the past 24 hours evaluated on an 11-stage rating scale from 0 [no pain] to 10 [maximum pain]) from the baseline was 1.61 in the 300 mg/day group and 2.33 in the 600 mg/day group. Although the target disease is different from that in this study, the evaluation criterion is similar to that in this study; that is, the reduction of the NRS score by 2 points or more is considered to be clinically relevant. Therefore, the proportion of subjects who meets the criterion will be examined in this study.

(3) EQ-5D-5L assesses each of the 5 health states including mobility, self-care, usual activities, pain/discomfort, and anxiety/depression using a 5-level scale and convert the results to one of 3125 QOL values (the fifth power of 5) to compare the change in QOL.

## Other Endpoints

1. Comparison between each dose and placebo groups (primary and secondary endpoints)
2. The relationship between the blood total phenytoin concentration and NRS scores will be examined to explore the effective concentration range.

<Rationale>

(1) This criterion was set to evaluate the relationship between dosage and analgesic effect.

(2) See <Rationale for the dosage> in Section 3.3.

## Safety Endpoints

The safety profile of the following endpoints will be compared among the groups (high-dose group, low-dose group, and placebo group).

1. Incidence of adverse events and adverse drug reactions
2. Incidence of serious adverse events and adverse drug reactions and narratives of individual events
3. Laboratory tests (hematology, blood biochemistry, and urinalysis)
4. Incidence of abnormal findings of blood pressure, heart rate, respiratory rate, electrocardiogram, and SpO_2_, as measured with biological information monitor

<Rationale>

(1) to (3) General items investigated in clinical studies

(4) Because NPC-06 has a risk of causing cardiac rhythm disorder when administered as a rapid intravenous bolus, subjects will be monitored using a biological information monitor from immediately before to 120 minutes after the start of the study treatment to assure safety. It will be examined whether or not the infusion rate affects cardiac rhythm.

# OBSERVATION/EXAMINATION/EVALUATION ITEMS AND TIMING

The investigators will observe, examine, or evaluate the following items and enter the results into the EDC. The study schedule is outlined in Table 10-1.

Table 10-1 Study Schedule

| Timing  Item | | | Screening ^*1^ | Investigational product administration day | | | | | | | | | | | Post-treatment observation period | | | | | Day of discontinuation |
| --- | --- | --- | --- | --- | --- | --- | --- | --- | --- | --- | --- | --- | --- | --- | --- | --- | --- | --- | --- | --- |
|  |  |  |  | Day 1 (day of administration) | | | | | | | | | | | Day2 | | | Day3 to 6 | Day7 |  |
|  |  |  |  | 120 minutes before the  start of administration | Immediately before the  start of administration | End of administration | After the start of administration | | | | | | | At bedtime | At the time of awakening | 24 hours after the start of administration | At bedtime | At bedtime | 6 days after the start of administration | At discontinuation |
|  |  |  |  | Before the  administration  of non-opioid  analgesics | 120 minutes  after the  administration  of non-opioid  analgesics |  | 30 minutes | 60 minutes | 90 minutes | 120 minutes | 4 hours | 6 hours | 8 hours |  |  |  |  |  |  |  |
| Acceptable time window^※1^ | | | On and after the day before administration | ± 10 minutes | -^※2^ | - | ± 5 minutes | ± 10 minutes | ± 10 minutes | ± 10 minutes | ± 15 minutes | ± 15 minutes | ± 30 minutes | - | - | ± 3 hours | - | - | ± 2 days | - |
| Informed consent ^*2^ | | | ● |  |  |  |  |  |  |  |  |  |  |  |  |  |  |  |  |  |
| Eligibility check | | | ● |  | ● |  |  |  |  |  |  |  |  |  |  |  |  |  |  |  |
| Subject registration | | |  |  | ● |  |  |  |  |  |  |  |  |  |  |  |  |  |  |  |
| Demographic and other characteristics | | | ● |  |  |  |  |  |  |  |  |  |  |  |  |  |  |  |  |  |
| Medical examination | | | ● | ● | ● |  |  |  |  | ● |  |  |  |  |  | ● |  |  | ● | ● |
| Height and body weight | | | ● |  |  |  |  |  |  |  |  |  |  |  |  |  |  |  |  |  |
| Vital signs | | | ● |  |  |  |  |  |  |  |  |  |  |  |  | ● |  |  | ● | ● |
| Biological information monitor ^*3^ | | |  |  |  |  |  |  |  |  |  |  |  |  |  |  |  |  |  |  |
| Study treatment^*4^ | | |  |  |  |  |  |  |  |  |  |  |  |  |  |  |  |  |  |  |
| Administration of non-opioid analgesics^*5^ | | | ● | ● |  |  |  |  |  |  |  |  |  |  |  |  |  |  |  |  |
| Patient diary  Efficacy evaluation | NRS | Evaluation point^*6^ | ● | ● | ● |  | ● | ● | ● | ● | ● | ● | ●^*8^ | ●^*9^ | ● | ● |  |  | ● | ●^*12^ |
|  |  | Mean^*7^ |  | ● |  |  |  |  |  |  |  |  |  |  |  |  |  |  |  |  |
|  |  | Maximum |  |  |  |  |  |  |  |  |  |  |  |  |  |  | ●^*10^ | ●^*11^ |  |  |
|  | EQ-5D-5L | | ● |  | ● |  |  |  |  | ● |  |  |  | ● |  | ● | ● | ● | ● | ●^*12^ |
| Laboratory tests | | | ●^*13^ |  |  |  |  |  |  |  |  |  |  |  |  | ● |  |  | ● | ● |
| Pregnancy test ^*14^ | | | ● |  |  |  |  |  |  |  |  |  |  |  |  |  |  |  | ● | ● |
| Blood drug concentration | | | ● |  |  |  | ● |  |  | ● |  |  |  |  |  | ● |  |  |  |  |
| Adverse events | | |  |  |  |  |  |  |  |  |  |  |  |  |  |  |  |  |  | ● |
| Concomitant drugs/therapies | | |  |  |  |  |  |  |  |  |  |  |  |  |  |  |  |  |  | ● |

※1: The starting point of the acceptable time window after the study treatment is the start time of administration.

※2: Perform evaluation and observation items at 120 minutes ±10 minutes after the administration time of non-opioid analgesics and perform subject registration.

*1: Perform screening on the day before administration or at least 120 minutes before the start of the study treatment on Day 1 (before the administration of non-opiod analgesics).

*2: Obtain written consent before starting the screening test.

*3: Continuously monitor blood pressure, heart rate, respiratory rate, electrocardiogram, and SpO_2_ on the biological information monitor.

*4: Start administering the investigational product within 60 minutes after subject registration as a rough guide and by 15:00 on Day 1 (day of administration).

*5: In principle, use the same non-opioid analgesic(s) during the period from the screening test to the end of evaluation at 24 hours after the start of the study treatment on Day 2.

*6: Record the pain intensity at the evaluation point using the NRS.

*7: Record the mean pain intensity over the past 24 hours using the NRS.

*8: Perform the evaluation at 8 hours after the start of administration as much as possible.

*9: If pain worsens after bedtime on Day 1, evaluate it according to the NRS and investigate the drug(s) used.
Subjects who waken at night for reasons other than the worsening of pain should evaluate the cause according to the NRS as much as possible.

*10: At bedtime on Day 2: Record the maximum pain intensity after the evaluation at 24 hours after the start of the study treatment using the NRS.

*11: At bedtime from Day 3 to Day 6: Record the maximum pain intensity over the past 24 hours using the NRS.

*12: Perform only when discontinued within 24 hours after the start of administration.

*13: If laboratory tests are performed by 3 days before Day 1, the data may be adopted.

*14: To be performed only for women of childbearing potential.

## Observation, Examination, and Evaluation Items at Screening

After obtaining the written consent to participate in the study from potential subjects, the investigators will check the following items by the start of the study treatment. Subjects will be hospitalized on the day before the study treatment or by 120 minutes before the start of the study treatment on the day of administration (Day 1). The investigators or clinical research coordinator will hand out the patient diary to subjects and instruct them on how to use it. The subjects will perform an NRS before blood collection.

| 1. Eligibility check based on the inclusion and exclusion criteria 2. Inquiry about demographic and other characteristics of subjects  - Race, sex, and date of birth - Diagnosis of herpes zoster (date and time of onset and site of onset of pain associated with acute herpes zoster and rash) - Type of rash caused by herpes zoster and proportion of crust over the whole rash - Type of pain associated with acute herpes zoster - Complications and medical history (medical history considered to affect drug efficacy evaluation within 1 year before registration [including surgical history]) - Prior medications/therapies  1. Medical examination   Subjective symptoms and objective findings   1. Height and body weight 2. Vital signs (blood pressure, pulse rate, and respiratory rate) 3. NRS (patient diary): Evaluation point 4. EQ-5D-5L (patient diary): Evaluation point 5. Laboratory tests^※^ 6. Pregnancy test (for women of childbearing potential) 7. Blood drug concentration 8. Administration of non-opioid analgesics (when not administered) |
| --- |

※ If laboratory tests are performed by 3 days before Day 1, the data may be adopted.

## Observation, Examination, and Evaluation Items during the Study Treatment Period

### Day of administration (Day 1)

#### At 120 minutes before the start of the study treatment

The investigators will check the following items at 120 minutes (± 10 minutes) before the start of the study treatment.

| The investigators will check the following items and administer a non-opioid analgesic at 120 minutes before the start of the study treatment. In addition, NRS score will be performed before the administration of a non-opioid analgesic.   1. Medical examination 2. NRS (patient diary): Evaluation point and mean 3. Prior medications/therapies 4. Administration of non-opioid analgesics |
| --- |

#### Immediately before the start of the study treatment (baseline)

The investigators will check the following items at 120 minutes (± 10 minutes) after the administration of a non-opioid analgesic.

| The investigators will check the following items and administer the investigational product.   1. Eligibility check based on the inclusion and exclusion criteria 2. Medical examination   Examination of subjects for ataxia, nystagmus, and dyslalia   1. NRS (patient diary): Evaluation point 2. EQ-5D-5L (patient diary): Evaluation point 3. Prior medications/therapies 4. Subject registration 5. Wearing of biological information monitor (blood pressure, heart rate, respiratory rate, electrocardiogram, and SpO_2_) |
| --- |

The investigators will enter the following records on the use of the investigational product into the EDC.

- Time to start and finish the administration of the investigational product (if discontinued, time to discontinue it)
- Dose based on body weight

#### Start of the study treatment

The investigational product will be administered by 15:00.

| - 1. Prepare the investigational product based on the body weight at the screening, dilute it 3 to 4-fold with physiological saline for injection, set it on an infusion stand or infusion pump, and fill the drug solution into the infusion line to the tip.   2. Secure the patient's intravenous line.   3. Administer the drug solution by drip infusion over at least 18 minutes in the high-dose group and over at least 12 minutes in the low-dose group.   4. Monitor the patient with the biological information monitor.   5. Record the time to start and finish the administration of the investigational product (if discontinued, time to discontinue it). |
| --- |

#### At 30 minutes after the start of administration of the investigational product

The investigators will check the following items at 30 minutes (± 5 minutes) after starting the administration of the investigational product. The subjects will perform an NRS before blood collection.

| 1. Biological information monitor (under continuous monitoring) 2. NRS (patient diary): Evaluation point 3. Blood drug concentration 4. Adverse events 5. Concomitant drugs/therapies |
| --- |

#### At 60 minutes after the start of administration of the investigational product

The investigators will check the following items at 60 minutes (± 10 minutes) after starting the administration of the investigational product.

| 1. Biological information monitor (under continuous monitoring) 2. NRS (patient diary): Evaluation point 3. Adverse events 4. Concomitant drugs/therapies |
| --- |

#### At 90 minutes after the start of administration of the investigational product

The investigators will check the following items at 90 minutes (± 10 minutes) after starting the administration of the investigational product.

| 1. Biological information monitor (under continuous monitoring) 2. NRS (patient diary): Evaluation point 3. Adverse events 4. Concomitant drugs/therapies |
| --- |

#### At 120 minutes after the start of administration of the investigational product

The investigators will check the following items at 120 minutes (± 10 minutes) after starting administration of the investigational product. The subjects will perform an NRS before blood collection.

| 1. Medical examination   Examination of subjects for ataxia, nystagmus, and dyslalia   1. Biological information monitor 2. NRS (patient diary): Evaluation point 3. EQ-5D-5L (patient diary): Evaluation point 4. Blood drug concentration 5. Adverse events 6. Concomitant drugs/therapies 7. Removal of the biological information monitor |
| --- |

#### At 4 hours after the start of administration of the investigational product

The investigators will check the following items at 4 hours (± 15 minutes) after starting the administration of the investigational product.

| 1. NRS (patient diary): Evaluation point 2. Adverse events 3. Concomitant drugs/therapies |
| --- |

#### At 6 hours after the start of administration of the investigational product

The investigators will check the following items at 6 hours (± 15 minutes) after starting the administration of the investigational product.

| 1. NRS (patient diary): Evaluation point 2. Adverse events 3. Concomitant drugs/therapies |
| --- |

#### At 8 hours after the start of administration of the investigational product

The investigators will check the following items at 8 hours (± 30 minutes) after starting the administration of the investigational product.

| 1. NRS (patient diary): Evaluation point 2. Adverse events 3. Concomitant drugs/therapies |
| --- |

#### At bedtime

Subjects will check the following items at bedtime.

| 1. NRS (patient diary): Evaluation point 2. EQ-5D-5L (patient diary): Evaluation point 3. Adverse events 4. Concomitant drugs/therapies |
| --- |

* If pain occurs after bedtime, evaluate it according to the NRS and investigate the drug(s) used. Subjects who waken at night for reasons other than the worsening of pain should evaluate the cause according to the NRS as much as possible.

## Observation, Examination, and Evaluation Items during the Post-treatment Observation Period

### Next day of the administration of the investigational product (Day 2)

#### At the time of awakening

Subjects will check the following items at the time of awakening.

| 1. NRS (patient diary): Evaluation point 2. Adverse events 3. Concomitant drugs/therapies |
| --- |

#### At 24 hours after the start of administration of the investigational product

The investigators will check the following items at 24 hours (± 3 hours) after starting the administration of the investigational product. The subjects will perform an NRS before blood collection. Subjects may be discharged after all evaluations are completed.

| 1. Medical examination   Examination of subjects for ataxia, nystagmus, and dyslalia   1. Vital signs (blood pressure, pulse rate, and respiratory rate) 2. NRS (patient diary): Evaluation point 3. EQ-5D-5L (patient diary): Evaluation point 4. Laboratory tests 5. Blood drug concentration 6. Adverse events 7. Concomitant drugs/therapies |
| --- |

#### At bedtime

Subjects will fill out the patient diary at bedtime.

| 1. NRS (patient diary): Maximum pain intensity after the evaluation at 24 hours after starting the administration of the investigational product 2. EQ-5D-5L (patient diary): Evaluation point 3. Adverse events 4. Concomitant drugs/therapies |
| --- |

### Two days after administration (Day 3) or later

#### At bedtime (Day 3 to Day 6)

Subjects will fill out the patient diary at bedtime.

| 1. NRS (patient diary): Maximum pain intensity in the past 24 hours 2. EQ-5D-5L (patient diary): Evaluation point 3. Adverse events 4. Concomitant drugs/therapies |
| --- |

### Six days after the start of administration of the investigational product (Day 7)

The investigators will check the following items at 6 days (± 2 days) after starting the administration of the investigational product. The investigators will collect the patient diary from the subjects.

| 1. Medical examination   Examination of subjects for ataxia, nystagmus, and dyslalia   1. Vital signs (blood pressure, pulse rate, and respiratory rate) 2. NRS (patient diary): Evaluation point 3. EQ-5D-5L (patient diary): Evaluation point 4. Laboratory tests 5. Pregnancy test (for women of childbearing potential) 6. Adverse events 7. Concomitant drugs/therapies |
| --- |

### At discontinuation

The investigators will check the following items for subjects who discontinued the study. The investigators will promptly enter the date or reason for discontinuation into the EDC. The investigators will record the time of discontinuation in the EDC for subjects who withdraw from the study after the administration of the investigational product on Day 1.

| 1. Medical examination   Examination of subjects for ataxia, nystagmus, and dyslalia   1. Vital signs (blood pressure, pulse rate, and respiratory rate) 2. NRS (patient diary): Evaluation point (only within 24 hours after the start of administration) 3. EQ-5D-5L (patient diary): Evaluation point (only within 24 hours after the start of administration) 4. Laboratory tests 5. Pregnancy test (for women of childbearing potential) 6. Adverse events 7. Concomitant drugs/therapies |
| --- |

# EFFICACY EVALUATION

## NRS

The evaluation using the NRS score will be performed for the site of pain associated with acute herpes zoster. It will be performed at the site of the most severe pain when there is more than one painful site. The pain intensity of the same site will be evaluated throughout the study period.

Each subject will assess pain severity using the 11-stage rating scale from 0 (no pain) to 10 (maximum pain) (NRS), and record the assessment site and pain intensity in the patient diary and record the evaluation site and pain severity in the patient diary as specified in 10 OBSERVATION/EXAMINATION/EVALUATION ITEMS AND TIMING and the study schedule (Table 10-1).

The investigators will instruct subjects how to evaluate pain.

Numerical Rating Scale (NRS)

Maximum pain

No pain

## EQ-5D-5L

Each subject will evaluate the quality of life using EQ-5D-5L (Patient Diary) as specified in 10 OBSERVATION/EXAMINATION/EVALUATION ITEMS AND TIMING and the study schedule (Table 10-1). He/she will describe the 5 health states including mobility, self-care, usual activities, pain/discomfort, and anxiety/depression using a 5-level scale. The sponsor will calculate QOL value using the evaluation result (5^5^ = 3125) and conversion table (tariff). The investigators will instruct subjects how to evaluate QOL.

# SAFETY EVALUATION

## Physical Examination

Subjective symptoms and objective findings will be observed at the screening.

Each subject will be examined for [1] ataxia, [2] nystagmus, and [3] dyslalia using the method listed in the table below to check the presence/absence of adverse events related to cerebellar symptoms ^14)^ at the clinical examinations during the period from immediately before the start of administration on Day 1 to the post-treatment observation period (or discontinuation). The investigators will choose appropriate examination(s)/test(s) at their own discretion.

Examinations of ataxia, nystagmus, and dyslalia

| [1] Ataxia | - Finger-nose test  - Finger-finger test  - Finger-nose-finger test  - Mann test  - Tandem gait  - Toe finger test  - Heel knee test  The above tests should be combined appropriately.  These tests should be performed to the extent possible, depending on patient's condition. |
| --- | --- |
| [2] Nystagmus | Gaze nystagmus test |
| [3] Dyslalia | Use the word list for single-word intelligibility test for evaluating speech of adults with articulation disorders^15)^. |

## Laboratory Tests

Laboratory tests with the following hematology, blood biochemistry test, and urinalysis parameters will be performed according to 10 OBSERVATION/EXAMINATION/EVALUATION ITEMS AND TIMING and the study schedule (Table 10-1). If part of the scheduled tests are neglected and performed on another date, record the test items, date of blood collection, date of urine collection, and reason.

| Hematology | Red blood cell count, white blood cell count, differential white blood count (basophil, eosinophil, neutrophil, lymphocyte, and monocyte), platelet count, hemoglobin, and hematocrit |
| --- | --- |
| Blood biochemistry | Total protein, albumin, AST (GOT), ALT (GPT), γ-GTP, Al-P, LDH,  total bilirubin, total cholesterol, HDL-cholesterol, triglyceride, BUN, creatinine, uric acid, Ca, P, Na, K, and Cl |
| Urinalysis | Protein, glucose, urobilinogen, and occult blood |

## Vital Signs

Blood pressure, pulse rate, and respiratory rate will be measured as specified in 10 OBSERVATION/EXAMINATION/EVALUATION ITEMS AND TIMING and the study schedule (Table 10-1). Body position (e.g., sitting or supine position) and measurement method (e.g., manual or machine) should not be changed throughout the study period.

## Biological Information Monitor

Blood pressure, heart rate, respiratory rate, ECG, and SpO_2_ will be continuously monitored with the biological information monitor from immediately before to 120 minutes after the start of the study treatment.

<Rationale>

The strict monitoring was specified because NPC-06 may cause circulatory and respiratory disorders, such as cardiac arrest, transient reduction in blood pressure, and respiratory depression, when it is administered as a rapid intravenous bolus.

## Pregnancy Test

Urinary hCG (qualitative) will be measured for female subjects. This test may be omitted for subjects who have passed 12 months since menopause, those undergoing menstruation, or those who have undergone the removal of the uterus or both ovaries.

<Rationale>

It was specified that each female subject should be asked about pregnancy status at the start and end of the study because the safety of NPC-06 during pregnancy has not been established.

## Adverse Events

### Definitions of adverse events

An adverse event is defined as any unfavorable or unintended medical sign (including abnormal laboratory findings), symptom, or disease that occurs during the period from the start of the study treatment to 6 days ± 2 days after the last dose (Day 7), irrespective of the causal relationship with the investigational product.

1. Onset or worsening of subjective symptoms or objective findings

1) New symptoms, findings, or diseases not observed before the start of the study treatment

2) Symptoms, findings, and diseases observed since before the start of the study treatment and worsened

1. Abnormal variations in laboratory test values

Any laboratory test value deviating from the range of the reference value. Abnormal variation is defined as follows.

1) Deviation from the reference values after the start of the study treatment

2) Deviation from the reference values worsened as compared to the value obtained immediately before the start of the study treatment

Any variation in laboratory test parameters that meets either of the above definitions will be handled as abnormal variation and adverse event, except clinically irrelevant variations. In this regard, however, any variation meeting the reference value may be handled as an adverse event when it is judged abnormal by the investigators.

### Severity of adverse events

The severity of adverse events will be judged with reference to the Common Terminology Criteria for Adverse Events v 4.0 (CTCAE) Japanese translation (JCOG version). For events not included in CTCAE, severity will be judged with reference to the following criteria.

| Grade 1 | Mild  Asymptomatic or mild symptoms  Clinical or diagnostic observations only  Intervention not indicated |
| --- | --- |
| Grade 2 | Moderate  Minimal, local or noninvasive intervention indicated  Limiting age-appropriate instrumental activities of daily living |
| Grade 3 | Severe or medically significant but not immediately life-threatening  Hospitalization or prolongation of hospitalization indicated  Disabling  Limiting selfcare activities of daily living |
| Grade 4 | Life-threatening consequences  Urgent intervention indicated |
| Grade 5 | Death related to adverse event |

### Significant adverse events

Significant adverse events are defined as adverse events that do not fall in the category of serious adverse events and are considered to be of special interest from the viewpoint of clinical significance. Events falling under any of the following categories will be handled as significant adverse events.

- - 1. Any respiratory or cardiac conduction adverse event for which the infusion of the investigational product was discontinued or should have been discontinued
    2. Any psychological/neurological adverse event for which the infusion of the investigational product was discontinued or should have been discontinued
    3. Adverse events leading to discontinuation of the investigational product

### Causal relationship with the investigational product

The causal relationship with the investigational product will be assessed with reference to the following criteria. Adverse events assessed as “related” will be considered to be “adverse reactions”.

| Not related | Clinical events including abnormal laboratory values that do not follow a reasonable temporal sequence after the administration of the investigational product and are considered to be due to, for example, a concurrent illness, another drug, or environmental factor. |
| --- | --- |
| Related | Events other than events judged "not related" |

### Observation of adverse events

The investigators will carefully monitor subjects for the onset of adverse events by interview and medical examination from the start of the study treatment to the post-treatment observation day or discontinuation. If any adverse event occurs, the investigators will give appropriate treatment as needed and follow the event as much as possible until it is resolved.

For all adverse events that occur, the name of the event, date of onset (or date and time when the event was found), severity, seriousness status (serious/non-serious), treatment status (specify if given), outcome, date of outcome evaluation, and causal relationship with the investigational product will be closely recorded in the EDC.

### Follow-up investigation and period after the onset of adverse events

1. The investigators will appropriately follow each adverse event and record the following items.

- Date of follow-up investigation

- Outcome (1. Recovered, 2. Recovering, 3. Not recovered, 4. Recovered with sequelae, 5. Death, 6. Unknown)

- Reason for the inability to follow an adverse event until recovery or recovery to the pretreatment status or to the status before onset, for the decision of the investigators to discontinue the follow-up, or for the refusal of a subject to follow-up.

1. For adverse events that do not recover or do not recover to the pre-onset status, the investigators will follow the event for approximately 3 weeks after the post-treatment observation day. The decision to further continue follow-up will be left to the investigators, although the investigators will continue follow-up when it is deemed necessary, as judged by the consultation of the sponsor with the medical expert. For irreversible adverse events by organic disorders, the investigators will follow them until the symptoms/findings become stable or fixed.

## Blood Drug Concentration Measurement

Blood samples will be collected as specified in 10 OBSERVATION/EXAMINATION/EVALUATION ITEMS AND TIMING and the study schedule (Table 10-1) to determine (1) blood total fosphenytoin concentration, (2) blood total phenytoin concentration, (3) blood free fosphenytoin concentration, and (4) blood free phenytoin concentration.

The samples will be analyzed by an external testing laboratory using LC/MS/MS, which will report the measurements after data fixation. In addition, the sponsor will report the measurements to each study site after unblinding.

# DISCONTINUATION CRITERIA AND PROCEDURES

## Discontinuation Criteria

The investigators will discontinue the study for subjects found to meet any of the following criteria after registration.

1. Continuous observation of any of the following findings by the biological information monitor

- Systolic blood pressure reduced by more than 20 mmHg from the value immediately before administration
- Diastolic blood pressure reduced by more than 10 mmHg from the value immediately before administration
- Heart rate reduced to less than 50

If any of the above criteria is met, the investigators will repeat measurement as necessary (any measurement method may be used). Subjects who met any of the above criteria may continue the study only when the investigators permit it based on the remeasurement results.

1. The investigators determined that the subject should be withdrawn from the study due to an adverse event.
2. The subject requested to discontinue the study.
3. Any prohibited concomitant drug or therapy was used.
4. The subject was found to meet any of the exclusion criteria during the study period.
5. The subject was found inappropriate for the study.
6. It was found that the subject could not receive essential observations or examinations for personal reasons.
7. Other cases where the investigators decide that the investigational product should be discontinued.

<Rationale>

(1)(2) These criteria were set to ensure safety.

(3) This criterion is included in the explanation given at the acquisition of informed consent and was set as a subject's right.

(4) This criterion was set because it was considered impossible to perform appropriate evaluation under the circumstance.

(5)(6) These criteria were set because subjects who should not be treated with the investigational product should be withdrawn from the study promptly.

(7) This criterion was set because subjects who could not undergo necessary observations should be withdrawn from the study promptly.

(8) The criterion was set assuming the cases where the study should be discontinued at the discretion of the investigators for other reasons.

## Discontinuation Procedures

The investigators will immediately discontinue the investigational product in subjects found to meet any of the discontinuation criteria during the study treatment. Subjects withdrawn from the study after the start of the study treatment should be requested to receive specified laboratory tests at discontinuation (see 10.3.4 At discontinuation) to confirm safety. Subjects withdrawn from the study should be given appropriate alternative treatment. For subjects with reduced blood pressure or heart rate meeting the criterion (1) of 13.1 Discontinuation Criteria after the dose of the investigational product on Day 1, the efficacy items specified until 120 minutes after the start of the study treatment should evaluated as much as possible and then the tests specified at discontinuation performed if they can be performed as judged by the investigators.

1. For subjects withdrawn from the study due to safety problems, such as the onset of adverse events:

Give appropriate treatment and follow them to the extent possible until symptoms recover to the baseline or acceptable level. Follow 14 SECURITY OF SAFETY OF SUBJECTS for subjects withdrawn from the study due to serious adverse events.

1. For subjects who requested to withdraw from the study:

Ask the reason for the request, respecting the rights of subjects.

1. For discontinuation or suspension of the study by the decision of the investigator for reasons, such as suspected safety of the investigational product:

Promptly notify the head of each study site of the discontinuation/suspension in writing and then explain the details in writing. The head of each study site will promptly report the discontinuation/suspension and reason to the IRB and sponsor in writing.

1. For subjects lost to follow-up:

Contact subjects with available means to ask the reason for not visiting and onset of adverse events, if any.

For subject who discontinued the study, the investigation of the process to discontinuation, date of discontinuation (date when the investigators determined discontinuation), reason for discontinuation, treatment given, and subsequent clinical course will be recorded, and the date of discontinuation and reason for discontinuation will be entered into the EDC.

# SECURITY OF SAFETY OF SUBJECTS

## Actions to Serious Adverse Events

### Definition of serious adverse events

Adverse events that meet any of the following criteria are considered to be a serious adverse event.

1. Death
2. Events that may lead to death
3. Events that require hospital admission or prolongation of existing hospital admission for treatment
4. Disability
5. Events that may lead to disability
6. Events that are as serious as (1) to (5)
7. Congenital disease or anomaly in later generations

For hospital admission and prolongation of existing hospital admission, a hospital admission that meets any of the following criteria does not apply: hospital admission specified in the clinical study protocol, hospital admission for scheduled surgery or detailed examinations, educational admission, hospital admission or prolongation of hospital admission for the convenience of subjects or caregivers, hospital admission for disease management, and hospital admission for health control (checkup).

### Handling of serious adverse events

1. Investigator

The investigators give appropriate treatment to subjects who develop serious adverse events. The investigators may request the opening of the emergency code when it is deemed necessary to treat serious adverse events (unblinding should be performed in accordance with 5.3.3 Unblinding procedure during the study).

The investigators will report serious adverse events according to the following procedure.

1. Immediately report the onset of a serious adverse event to the head of the study site and sponsor.
2. Prepare a Serious Adverse Event Report as early as possible within 7 days after the report to the sponsor, submit it to the sponsor and head of the study site, and keep a copy.
3. Try to collect more detailed data and, if any is collected, report them to the sponsor and head of the study site.

| Emergency contact (Sponsor) |
| --- |
| Nobelpharma Co., Ltd.:  1-17-24 Shinkawa, Chuo-ku, Tokyo 104-0033  TEL: 03-6670-3811, FAX: 03-6670-5051  E-mail address: arai@nobelpharma.co.jp  < Nighttime (18:00 to 9:00) and all day on Saturdays, Sundays, and holidays>  Saori Arai (mobile phone: 080-6842-3235)  Kenji Shimizu (mobile phone: 090-1588-3296) |

1. Sponsor

The sponsor will follow the following procedure in receiving a report on the onset of a serious adverse event from the sponsor.

1. Obtain the Serious Adverse Event Report as soon as possible within 7 days after the receipt of the report from the investigator.
2. Obtain the advice from the medical expert about the seriousness and causal relationship of the event with the investigational product.
3. Submit the report to the competent authorities within the specified deadline if the event falls under the provisions of Article 273 of the Enforcement Regulations of the Pharmaceutical and Medical Device Act. Immediately report the adverse event to each investigator and head of the study sites in writing.

## Pregnancy

The investigators will immediately withdraw subjects found or suspected to be pregnant during the study period. The investigators will report to the sponsor the designated information at a minimum, including the subject identification code, date of onset, date of investigational product administration, dose, and date of discontinuation. If the partner of a subject is found or suspected to become pregnant, the investigators shall obtain the information on the pregnancy with the approval of the partner. Although pregnancy is not handled as an adverse event, the investigators will follow pregnancy until outcome (e.g., delivery) is known.

# CLINICAL STUDY PROTOCOL COMPLIANCE, DEVIATION OR CHANGE, AND REVISION

## Clinical Study Protocol Compliance

1. This study will be conducted in compliance with the clinical study protocol based on the agreement between the investigator and sponsor.
2. The investigators must not deviate from or change the clinical study protocol without prior written agreement with the sponsor and prior approval of the IRB. However, this does not apply to medically unavoidable cases to avoid an immediate danger to subjects (hereinafter referred to as an emergency deviation).

## Clinical Study Protocol Deviations or Modifications

1. In the case of an emergency deviation, the investigator will submit a report on the deviation from the clinical study protocol to avoid an immediate danger to the sponsor and head of the study site and obtain approval from the IRB and head of the study site and agreement of the sponsor.
2. The investigators will record all deviations from the clinical study protocol, regardless of reasons.

## Clinical Study Protocol Amendment

1. The sponsor must revise the clinical study protocol in consultation with the medical expert as necessary when:

- Information related to the quality, efficacy, or safety of the test drug or other important information for the proper conduct of the clinical trial is known; or
- It is necessary to revise the clinical study protocol for medically inevitable reasons.

1. The sponsor will promptly notify each investigator of the fixed revisions in writing.
2. The investigator will fully review the ethical and scientific validity of conducting the study according to the revised clinical study protocol.
3. The sponsor and investigator will discuss the revised clinical study protocol and sign and seal or sign 2 copies of the clinical study protocol or alternative document as a proof of agreement. The sponsor will then obtain the approval of the IRB.
4. If the IRB or clinical study secretariat instructs modifications, the sponsor will consult with the medical expert as appropriate and report the results to the investigators.

# COMPLETION, DISCONTINUATION, OR SUSPENSION OF THE STUDY

## Completion of the Study

The investigator will promptly enter the results in the EDC when the administration and observation specified in the clinical study protocol is completed for the last subject at the study site. The investigator will report the completion of the study and summary of the study results to the head of the study site in writing.

The head of the study site will promptly notify the IRB and sponsor of the completion of the study in writing and report the summary of the study results based on the report submitted by the investigator.

## Discontinuation or Suspension of the Study

1. Criteria for discontinuation or suspension

The sponsor will decide to discontinue or temporarily suspend the study through the consultation with the medical expert if any of the following criteria is met.

1) Occurrence of safety problems, such as a serious adverse drug reaction

2) Recommendation of the IRB to discontinue or temporarily suspend the study

3) Recommendation of the competent authorities to discontinue the study

4) Change of the development policy of the sponsor

5) Other situations requiring the discontinuation or suspension of part or all of the clinical study

1. Procedure for discontinuation or suspension

The sponsor will discontinue or temporarily suspend the study according to the following procedure if the sponsor deems it necessary to discontinue or temporarily suspend part or all of the study.

1. Upon the decision to discontinue or temporarily suspend the study, the sponsor will promptly notify the investigator, head of each study site, and regulatory authorities of the discontinuation or suspension and its reason in writing.
2. When the investigator is notified of discontinuation or suspension of the study by the sponsor, the investigators will promptly report it to the subjects and assure them of appropriate follow-up treatment.
3. To discontinue or temporarily suspend the development of the investigational product, the sponsor will promptly notify the investigator, head of each study site, and regulatory authorities of the fact and its detailed reasons in writing.

# CASE REPORT FORMS

## Format of the Case Report Form to Be Used in This Study

An EDC system (electronic case report form) conforming to the ER/ES guidelines will be used.

## Entry to and Reporting with Case Report Forms

The investigators and clinical research coordinators will enter the observation, examination, and evaluation data into the EDC based on source documents. The entry by clinical research coordinators is allowed only for the transcription of data described in source documents.

For the method of entry, change, and correction of the EDC, the Procedure for Preparation, Change or Correction of Electronic Case Report Forms provided separately by the sponsor should be followed.

In addition, only subinvestigators or clinical research coordinators registered in the List of Subinvestigators and Clinical Research Coordinators are allowed to perform the entry in the EDC.

The investigators and clinical research coordinators will be granted the access to the EDC system after receiving education and training. The sponsor (including the CRO's data management and monitors) will issue a query for inconsistency or question about input data and request the reconfirmation, addition, change, or correction of input data as necessary by data cleaning or SDV.

In this study, prior and concomitant medications will be coded according to the WHO Drug Dictionary. Similarly, the names of diseases will be coded with MedDRA preferred terms.

## Review of Case Report Forms by the Investigator

The investigator will check the entries by subinvestigators or clinical research coordinators after every entry or before data fixation. The investigator will check entries in the EDC, confirm that all entries (including audit trails and responses to queries) are accurate and complete, and guarantee them by writing his/her electronic signature.

For EDC data inconsistent with source documents, the investigator will prepare a record explaining the reason, appropriately retain it, and promptly submit a copy to the sponsor.

After locking the database, the investigator will receive a copy of the subject data from the sponsor and retain it at the study site.

## Preparation and Reporting of the Patient Diary

Subjects will record NRS and EQ-5D-5L in the patient diary.

The investigators will collect the patient diary at an appropriate timing, check the contents, and make inquiries to the subject as necessary. If there are any deficiencies, the subject will be requested to make additions/correction. Subjects will be instructed to correct entries using a double or single line so that the original entry will remain visible. If the investigator will make corrections, he/she will sign or seal, date, and write the reason for correction. All the patient diaries will be collected and stored before the completion (discontinuation) of the study. The sponsor will not collect the patient diary.

# SOURCE DOCUMENTS

## Identification of Source Documents

Source documents are records necessary for the reproduction and evaluation of the course of events in the clinical study. At the same time, source documents are documents, data and records that serve as the basis for case report forms. Specifically, source documents include the following:

1. Records concerning informed consent of subjects and provision of information to subjects
2. Records on the control of the investigational products
3. Subject screening and registration lists
4. Records that serve as the basis for case report forms, including medical records, nursing records, test/examination data, and patient diaries
5. Other documents or records related to this study

## Identification of Items for Which Data in Case Report Form Should Be Handled as Source Documents

If the following items are not described/entered in source documents, such as the medical record, the information directly entered into the EDC will be handled as source documents.

1. Reasons for use of prior/concomitant drugs and reasons for giving prior/concomitant therapies
2. Severity and seriousness of adverse events, causal relationship with the investigational product, and related descriptions
3. Description of judgment of abnormal changes in laboratory test values
4. Detailed description of follow-up of discontinued (dropout) cases
5. Study-related comments

## Direct Access to Source Documents

Based on the study contract, the head of each study site and investigator will accept direct access by the monitors and auditors of the sponsor, the IRB, and regulatory authorities for investigation and confirmation of the records the study site must retain, such as source documents and essential documents.

The method, timing, and items of the direct access by the sponsor will be determined through the consultation between the investigator/study site and sponsor.

# STATISTICAL ANALYSIS

Statistical analyses will be performed according to Statistical Principles for Clinical Trials (PMSB/ELD Notification No. 1047, dated November 30, 1998). The statistical items and technical details described in this section will be separately specified in the Statistical Analysis Plan, which will be prepared before unblinding.

## Analysis Populations

### Subjects included in the efficacy analysis

All enrolled subjects will be included in the full analysis set (FAS), excluding those who do not receive the investigational product and those for whom no efficacy information is obtained after the start of the study treatment.

All enrolled subjects evaluable for efficacy except those with significant clinical study protocol violations after the start of the study treatment will be included in the per-protocol set (PPS). Sensitivity and other analyses will be performed in the PPS, as required.

### Subjects included in the safety analysis

All the subjects who received the investigational product and provided any safety information will be included in the safety population (SP).

## Statistical Analysis Items and Methods

### Demographic and other baseline characteristics

For demographic data and endpoints in the screening before the study treatment, summary statistics will be calculated for continuous variables and frequency distributions (number of subjects, %) will be calculated for categorical variables by treatment group.

### Efficacy evaluation

#### Primary endpoint

The NPC-06 and placebo groups will be compared in the following analysis.

The data on the NRS score will be collected immediately before (baseline) and 30, 60, 90, and 120 minutes after the start of the study treatment, and slope, as defined as the mean score change in the NRS score over the evaluation period, will be compared between the groups.

[Statistical model, hypothesis, and analysis method]

The NRS score at the baseline and 30, 60, 90, and 120 minutes after the start of the study treatment in the FAS will be analyzed with the mixed effects model using subject as a random effect and treatment group (high-dose group vs. placebo group and low-dose group vs. placebo group), time point, and treatment group x time point (slope) as a fixed effect to compare the slope between the groups. Slope represents the mean score change per hour until 120 minutes. The analytical process will proceed according to the closed testing procedure in the order of [1] high-dose group vs. placebo group followed by [2] low-dose group vs. placebo group, considering multiplicity.

[Sensitivity analysis]

The same analysis will be performed in the PPS to examine the robustness of the primary endpoint.

#### Secondary endpoints

The NPC-06 and placebo groups will be compared in the following analysis.

1. Change in the NRS score at each evaluation point (4, 6, and 8 hours after the start of the study treatment, at bedtime, at the time of awakening, and 24 hours after the start of the study treatment) from the baseline

[Analytical method]

The change at each evaluation point after the administration of the investigational product (4, 6, and 8 hours after the start of administration of the investigational product, at bedtime, at the time of awakening, and 24 hours after the start of the study treatment) from the baseline will be compared between groups with a linear regression model using baseline as a covariate and treatment group (high-dose, low-dose, and placebo groups) as a fixed effect.

1. Proportion of subjects in whom the NRS score is improved by at least 2 points at 120 minutes after the start of the study treatment as compared to the score immediately before administration

[Analytical method]

The proportion of subjects in whom the investigational product is evaluated effective in each group (high-dose group, low-dose group, and placebo group) is examined using the Fisher’s exact test, considering the improvement of the NRS score by at least 2 points at 120 minutes after the start of the study treatment from the baseline as effective.

1. Change in QOL value of EQ-5D-5L

[Analytical method]

The change in the QOL value at each evaluation point after administration of the investigational product from the baseline is compared using the Wilcoxon rank sum test.

#### Other endpoints

The primary and secondary endpoints are compared between each dose and placebo group. The relationship between the blood total phenytoin concentration and NRS scores will be examined to explore the effective concentration range.

### Safety evaluation

The following analyses will be performed for the high-dose, low-dose, and placebo groups separately.

1. Adverse Events and Adverse Drug Reactions

Adverse events will be coded with MedDRA and tabulated by SOC and by PT. The number of subjects with events, incidence and number of events will be determined in the SP.

1. Other safety endpoints (general laboratory test parameters, biological information monitor, and vital signs)

Summary statistics will be calculated for continuous variables and frequency distributions (number of subjects, %) for categorical variables by observation time points in the SP.

### Pharmacokinetic analysis (PK/PD and biomarkers)

The change in the blood drug concentration at the screening and 30 and 120 minutes after the start of the study treatment will be graphically shown and summary statistics will be calculated by the treatment groups in the SP for the total phenytoin concentration, total fosphenytoin concentration, free phenytoin concentration, and free fosphenytoin concentration.

### Interval estimation and significance level

The significance level will be 5% (2-sided). The confidence coefficient (1-α) for interval estimation will be 95% (2-sided).

### Handling of missing data, unscheduled observations, and other unusual data

Missing data will not be imputed. Data not collected within the scheduled observation time window will not be used for tabulation and analysis at the time point. However, for subjects withdrawn from the study immediately after the administration of the investigational product, the data collected at each scheduled observation time window will be used for tabulation and analysis at the time point.

The handling of other data to be reviewed will be decided through the consultation of the sponsor with the medical expert before unblinding.

Data from subjects who withdraw their consent by themselves or their legally acceptable representative are not used for tabulation and analysis.

### Subgroup analyses

Stratified analyses will be performed as necessary.

### Exploratory analyses

The effects of background factors on endpoints will be analyzed in an exploratory manner as necessary. Summary statistics will be calculated, change diagrams will be prepared, and exploratory analyses of the primary and secondary endpoints between NPC-06 and placebo groups will be performed, as needed.

### Interim analysis

Not performed.

### Additions and changes to the statistical analysis plan

The details of the statistical methods will be provided in the statistical analysis plan. The statistical analysis plan will be finalized before database lock. If there is any change to the analysis plan described in the clinical study protocol, the analysis plan will be revised to describe the date and reason for the change(s).

# QUALITY CONTROL AND ASSURANCE OF THE STUDY

The sponsor will control and assure the quality of the clinical study using standard operating procedures to verify that the quality of the study is maintained.

## Quality Control

The sponsor will confirm whether the following main operations are appropriately performed according to each standard operating procedure.

1. In order to standardize the study methods, the sponsor or monitor will provide the investigators and other personnel involved in the conduct of the study with a thorough explanation of the methods of subject selection, enrollment, testing, and evaluation before starting the study.
2. The monitor(s) will periodically monitor study sites according to the monitoring procedure to confirm that the written consent from is obtained from subjects and the study is conducted in compliance with GCP, the clinical study protocol, and the investigational product control procedure.
3. The monitor(s) will collect information on adverse events during monitoring activities.
4. The monitor(s) will confirm the entries to the EDC based on the source documents, such as medical records.
5. The data management unit will control data quality according to applicable standard operating procedures of the sponsor and the data management plan established prior to the study. The data management unit will confirm the consistency, accuracy, and lack of logical errors for the data in the case report forms.
6. In the above (5), the data management unit will issue queries to correct deficiencies, ask questions, and request the investigators or clinical research coordinators to make a re-check, addition, change, or correction, as required. In addition, if the subinvestigators or clinical research coordinators make an addition, change, or correction, the investigator will ultimately review it.
7. The sponsor will review individual cases as necessary, verify the entries to the EDC, and examine the handling of the cases.
8. The data management manager and person in charge will perform computer data processing and reliability assurance according to the data management plan.
9. The sponsor will review the documents prepared, including the clinical trial plan notification, clinical trial request form/contract, and investigational product delivery/collection record.

## Quality Assurance

Auditors will assure the quality of this study according to the standard operating procedure and predetermined audit plan/procedure. Auditors will confirm that this study is conducted in compliance with the standard operating procedure, clinical study protocol, and related laws and regulations, such as Pharmaceutical and Medical Device Act and GCP. The sponsor, contract research organization, and study sites will be audited.

# ETHICAL CONDUCT OF THE STUDY

## Compliance with GCP

This study will be conducted in compliance with the ethical principles based on the Declaration of Helsinki, related laws and regulations such as provisions of Article 14, Paragraph 3 and Article 80, Paragraph 2 of the Act on Securing Quality, Efficacy and Safety of Pharmaceuticals, Medical Devices, Regenerative and Cellular Therapy Products, Gene Therapy Products, and Cosmetics (hereinafter referred to as the Pharmaceutical and Medical Device Act) and the Ministerial Ordinance on Good Clinical Practice (GCP), and this clinical study protocol.

## Patient Information Sheet

### Preparation of patient information sheet

Prior to the conduct of the study, the investigator will prepare the patient information sheet with the informed consent form in cooperation with the sponsor, submit it to the head of the study site to obtain approval from the IRB, and submit it to the sponsor.

The patient information sheet with the informed consent form shall contain the following contents specified in GCP.

1. That the study involves research
2. Objective of the study
3. Methods (including the experimental aspect of the study, subject inclusion criteria, and probability of random assignment to each treatment)
4. Expected duration of participation of subjects in the study
5. Planned number of subjects participating in the study
6. Expected clinical benefits and risks or inconvenience
7. Availability of other treatment methods and their expected significant benefits and risks
8. Compensation and treatments subjects can receive if any study-related injury occurs
9. That the subject's participation in the study is voluntary and that the subject and his/her legally acceptable representative may refuse to participate or withdraw from the study at any time. That the subject will not be disadvantaged by refusal or withdrawal or will not lose benefits to which the subject is otherwise entitled.
10. That the subject and the subject's legally acceptable representative will be informed in a timely manner if information becomes available that may be relevant to the subject's willingness to continue participation in the study.
11. The foreseeable circumstances and/or reasons under which the subject's participation in the study may be terminated.
12. That the monitors, auditors, the IRB, and regulatory authorities will have access to the original medical records. In that case, the subject's privacy will be protected. The subject and the subject's legally acceptable representative are considered to authorize such access by signing and sealing the informed consent form.
13. That the subject's privacy will remain confidential even if the results of the study are published.
14. The anticipated expenses, if any, to be borne by the subject for participating in the study
15. The anticipated prorated payment, if any, to the subject for participating in the study (e.g., agreement on the calculation of the amount of payment)
16. Name, title, and contact information of the investigator or subinvestigator
17. The person (s) to contact at the study site for further information regarding the study and rights of study subjects or in the event of study-related injuries
18. Rules to be followed by subjects
19. Type of the IRB that investigates and reviews the appropriateness of the study, matters to be investigated and reviewed by the IRB, and other matters related to the IRB involved in the study
20. That the procedure manuals of the IRB can be checked. That the procedures of the IRB are available for public viewing by providing the website address if the procedures of the IRB are disclosed on the website of the study site, or by providing the procedures of the IRB in the office if the procedures are not disclosed. That the procedures of the IRB can be reviewed upon the request.

### Timing and method of obtaining informed consent

#### Timing of obtaining informed consent

After the study contract is concluded between the study site and the sponsor and before tests/observations described in the clinical study protocol are performed, the investigators will explain the study to candidate subjects using the patient information sheet with the informed consent form approved by the IRB, give them opportunities to ask questions and ample time to determine whether or not to participate in the study, and obtain their voluntary written consent.

However, if the patient who is a candidate subject requires a representative, the patient's legally acceptable representative (a person who exercises parental power over the patient who is a candidate subject, guardian, or other equivalent person who can work for the patient's best interest) will be also provided with adequate explanation using the patient information sheet with the informed consent form, given opportunities to ask questions and ample time to determine whether or not to participate in the study, and then written voluntary consent will be obtained from the legally acceptable representative. At the same time, records will be kept showing the relationship between the legally acceptable representative and the candidate subject.

If the patient refuses to participate in the study, it is prohibited to make the patient participate in the study only with the consent of his/her legally acceptable representative.

#### Delivery of the informed consent form

After the explanation is completed, the investigators will sign the informed consent form, enter the date of explanation, and hand over it with the patient information sheet to candidate subjects. If a clinical research coordinator has provided supplementary explanation, the clinical research coordinator will also sign and date the consent form.

Before informed consent is obtained, the investigators will provide candidate subjects with opportunities to ask questions and ample time to determine whether to participate in the study. All questions must be answered to the satisfaction of candidate subjects.

#### Obtaining informed consent

Before performing the tests and observations necessary for the study, the investigators will obtain the consent form signed and dated by candidate patients for subject (if the candidate subject requires a representative, the patient and his or her legally acceptable representative) and give a copy to the subject along with the patient information sheet.

The original informed consent form will be retained at the study site. The investigators will denote in an appropriate document (e.g., the original informed consent form or medical record) that a copy of the informed consent form and patient information sheet were given to the subject.

### Revision of the patient information sheet

If new information becomes available that may affect the subject’s willingness to continue participation in the study, the investigator will revise the patient information sheet with the informed consent form based on the information and submit it to the IRB for approval.

The investigators will promptly inform subjects of the information, reconfirm their will as to whether or not to continue to participate in the study, and record the information in the medical record. Furthermore, the investigators will explain the revised patient information sheet approved by the IRB to subjects again and obtain a written re-consent as specified in 21.2.2 Timing and Method of Obtaining Informed Consent.

### Other matters

The investigators will comply with the following in obtaining consent from candidate patients.

1. The investigators must not exert undue influence on candidate patients: for example, the investigators must not force them to participate or continue to participate in the study.
2. The patient information sheet with the informed consent form and information provided orally to subjects must not contain any words or phrases that will or may make them give up their rights or relieve the investigators, study sites, or the sponsor from their legal responsibilities.
3. The patient information sheet with the informed consent form and orally provided information should use non-technical language that can be understood by candidate patients.
4. The study must not be initiated with oral consent alone for any reason.

## Review by the IRB

Prior to the conduct of the study, the IRB designated by each study site will obtain the clinical study protocol, informed consent form, investigator's brochure, and other necessary documents, review the conduct and continuation of the study from ethical, scientific, and medical viewpoints, and notify the head of the study site of his/her opinions in writing.

## Protection of Subjects' Human Rights

To select appropriate subjects, the investigators will carefully examine whether to ask for participation in the study from the viewpoint of protection of human rights, considering the health status, symptoms, age, ability to consent, dependency with the investigators, and participation in other clinical studies, as judged from the inquiry about the inclusion and exclusion criteria.

The investigators will give full consideration to the confidentiality of subjects. Only the subject identification code will be entered into the EDC. The name of subject, if any, in other documents/materials to be submitted to the sponsor will be deleted. Consideration will be given to the protection of privacy of subjects when the study results are published for academic purposes.

The sponsor, monitors, and auditors must not disclose any personal information obtained during the course of their duties to any third party. The same shall apply after retirement.

# RETENTION OF RECORDS

## Study Sites

The head of each study site will retain essential documents or records to be retained at the study site until the date specified in (1) or (2) below, whichever comes later. However, if the sponsor requires a longer retention period, the sponsor will consult with the study site about the retention period and method. Records will be retained by appointed persons in charge of record retention.

1. Date of the marketing approval of the test drug (if development is discontinued, the day 3 years after the date of discontinuation of development)
2. Day 3 years after the discontinuation or completion of the study

## The Founder of the Institutional Review Board

The founder of the IRB will retain the standard operating procedures, member list (including the qualification of each member), list of occupation and affiliation of members, submitted documents, meeting minutes and summary, and records, such as letters until the day of (1) or (2), whichever comes later. However, if the sponsor requires a longer retention period, the sponsor will consult with the study site about the retention period and method.

1. Date of marketing approval of the test drug (if development is discontinued, the date of receiving the notification of discontinuation of development)
2. Day 3 years after the discontinuation or completion of the study

## Sponsor

The sponsor will retain essential documents or records to be retained until the following (1) or (2), whichever comes later.

1. The day 5 years after the day of marketing approval for the test drug. However, materials related to drugs for which re-examination is required pursuant to the provisions of Article 14, Paragraph 4, Item 1 of the Pharmaceutical and Medical Device Act (limited to those for which the period from the date of approval to completion of re-examination exceeds 5 years) will be retained until completion of re-examination.
2. Day 3 years after the discontinuation or completion of the study

In addition, for materials to be stored by contractors, the sponsor will consult with contractors about the materials to be stored and the place and period of storage.

## Notification from the Sponsor after the End of the Storage Period

When essential documents or records retained by the head of each study site and the founder of the IRB become no longer necessary, the sponsor will notify the head of each study site or the founder of the IRB via the head of each study site of the fact.

# PAYMENT AND INSURANCE

## Compensation for Study-related Injuries and Insurance

When subjects experience any study-related injury attributable to this study, the study site will give treatment and take other necessary actions and the sponsor will provide appropriate compensation for the study-related injury, except in cases where the study-related injury is attributable to the intentional or serious fault of the study site or subjects. The sponsor will be liable for any study-related injury attributable to this study unless the injury is attributable to a specific study site. The sponsor will take out an appropriate insurance or take other necessary measures to legal liability/compensate for study-related injuries.

## Payment

Payments related to this study will be described in a separate agreement or contract between the sponsor and study sites.

# PUBLICATION POLICY

The investigators shall obtain prior written approval from the sponsor when they intend to publish the results obtained from the study in academic conferences or journals.

The sponsor has the copyright to the publications to be prepared or entrusted to be prepared based on the results of the study. The information contained in this clinical study protocol (particularly unpublished data) is also the property of the sponsor. Therefore, the investigators who are willing to participate in this study, persons involved in the conduct of the study, study sites, institutional review boards may not disclose such information provided by the sponsor to third parties without written consent of the sponsor, except when the information is used for obtaining consent from subjects.

The data obtained from this study will be the property of the sponsor and if part or all of the results of this study are published in academic conferences or medical journals (except the disclosure to regulatory authorities), prior approval of the sponsor is required.

The results of this study may be used for publications of the sponsor and for application materials to be submitted to regulatory authorities.

# STUDY ORGANIZATION

## Sponsor

### Sponsor

Jin Shiomura, President, Nobelpharma Co., Ltd.

1-17-24 Shinkawa, Chuo-ku, Tokyo 104-0033

TEL: 03-6670-3800, FAX: 03-6670-3801

### Clinical development manager

To manage and supervise the overall operations related to the clinical study and take full responsibility for smooth conduct of the clinical study.

Shigeki Shimasaki, Director of Research and Development Division, Nobelpharma Co., Ltd.

1-17-24 Shinkawa, Chuo-ku, Tokyo 104-0033

TEL: 03-6670-3800, FAX: 03-6670-3801

### Clinical study manager

To assume responsibility for the promotion and management of operations related to the clinical study. To direct and manage the operations contracted out to the contract research organization.

Saori Arai, Clinical Development Department 2, Nobelpharma Co., Ltd.

1-17-24 Shinkawa, Chuo-ku, Tokyo 104-0033

TEL: 03-6670-3811, FAX: 03-6670-5051

### Medical expert

To give instructions and advice to the sponsor on medical matters related to the clinical study from medical and technical viewpoints, including the advice on drafting and revision of the investigator's brochure, clinical study protocol, electronic case report form, and patient information sheet with informed consent form, instructions on the continuous evaluation of and actions to the safety information related to the investigational products, medical evaluation of case data obtained with the investigational product, and instructions on the preparation of the clinical study report.

Masako Iseki, Professor, Department of Anesthesiology and Pain Medicine, Juntendo University School of Medicine

### Coordinating investigator

To coordinate the interpretation and other details of the clinical study protocol between study sites involved in the multicenter clinical study.

Makoto Kawashima, Professor Emeritus, Tokyo Women's Medical University

### Monitor

To investigate the progress of the study in accordance with applicable standard operating procedures to confirm that the study has been conducted, recorded, and reported in accordance with the clinical study protocol, standard operating procedures, standards specified in Article 14, Paragraph 3 and Article 80, Paragraph 2 of the Pharmaceutical and Medical Device Act, and GCP.

Saori Arai, Clinical Development Department 2, Nobelpharma Co., Ltd.

1-17-24 Shinkawa, Chuo-ku, Tokyo 104-0033

TEL: 03-6670-3811, FAX: 03-6670-5051

### Data management manager

To manage the data management operations outsourced to the contract research organization.

Yuko Ishikawa, Data Science Office, Nobelpharma Co., Ltd.

1-17-24 Shinkawa, Chuo-ku, Tokyo 104-0033

TEL: 03-6670-3800, FAX: 03-6670-3801

### Statistical analysis manager

To manage the statistical analyses outsourced to the contract research organization.

Izumi Hamada, Data Science Office, Nobelpharma Co., Ltd.

1-17-24 Shinkawa, Chuo-ku, Tokyo 104-0033

TEL: 03-6670-3800, FAX: 03-6670-3801

### Quality control manager

To confirm that essential documents have been prepared based on GCP and standard operating procedures.

Yusaku Ishizuka, GCP Quality Control Office, Nobelpharma Co., Ltd.

1-17-24 Shinkawa, Chuo-ku, Tokyo 104-0033

TEL: 03-6670-3800, FAX: 03-6670-3801

### Audit manager

To contract out the audit activities to the contract audit organization and control the activities of the organization in accordance with applicable standard operating procedures of Nobelpharma Co., Ltd. The operations contracted out to the contract audit organization include the audit of the appropriateness of the clinical study system, appropriateness of the operations, and reliability of data at the sponsor, study sites, and contract research organization.

GCP Audit Office, Nobelpharma Co., Ltd.

1-17-24 Shinkawa, Chuo-ku, Tokyo 104-0033

### Investigational product control manager

To control the storage, delivery, collection, and disposal of the investigational products according to standard operating procedures and Investigational Product Handling Procedure of Nobelpharma Co., Ltd.

Masahito Hayashi, GCP Quality Control Office, Nobelpharma Co., Ltd.

1-17-24 Shinkawa, Chuo-ku, Tokyo 104-0033

TEL: 03-6670-3800, FAX: 03-6670-3801

## Contractor

### Contract research organization (CRO)

#### Monitoring

To investigate the progress of the study in accordance with applicable standard operating procedures to confirm that the study has been conducted, recorded, and reported in accordance with the clinical study protocol, standard operating procedures, standards specified in Article 14, Paragraph 3 and Article 80, Paragraph 2 of the Pharmaceutical and Medical Device Act, and GCP.

Noriko Kojima, Manager, Imepro, Inc.

4-8-16, Nihonbashi Honcho, Chuo-ku, Tokyo 103-0023

TEL: 03-6661-6623, FAX: 03-6661-6653

#### Data management

To prepare the data management plan, check data according to the plan, and fix data. To amend and fix electronic case report forms, as required, according to the separately specified procedure. To perform operations under the instruction of the sponsor by fully consulting with the person in charge of DM/BS from the sponsor.

Masayuki Ideta, Manager, Imepro, Inc.

4-8-16, Nihonbashi Honcho, Chuo-ku, Tokyo 103-0023

TEL: 03-6661-6623, FAX: 03-6661-6653

#### Statistical analysis

To prepare the statistical analysis plan in accordance with the analysis methods described in the clinical study protocol and perform analysis and tabulation according to the plan. To perform operations under the instruction of the sponsor by fully consulting with the person in charge of DM/BS from the sponsor.

Junichi Suzuki, Manager, Imepro, Inc.

4-8-16, Nihonbashi Honcho, Chuo-ku, Tokyo 103-0023

TEL: 03-6661-6623, FAX: 03-6661-6653

#### Audit

To confirm the appropriateness of the clinical study system, appropriateness of the operations, and reliability of data related to the clinical study and perform operations by consulting with the audit manager of the sponsor.

Toshitaka Kato, Manager, Imepro, Inc.

4-8-16, Nihonbashi Honcho, Chuo-ku, Tokyo 103-0023

TEL: 03-6661-6623, FAX: 03-6661-6653

#### Investigational product assignment manager

To prepare and manage the assignment table, assign the investigational products, confirm the indistinguishability of the package, prepare the emergency code, unblind the assignment table, and confirm the blindness of the investigational product before unblinding.

Junichi Yoshitake, Manager, Imepro, Inc.

4-8-16, Nihonbashi Honcho, Chuo-ku, Tokyo 103-0023

TEL: 03-6661-6623, FAX: 03-6661-6653

### Drug concentration measuring laboratories

To measure the concentration of specified drugs using blood drug concentration measurement samples collected at study sites and report the results to the sponsor. To retain residual samples and raw data including all the charts of LC/MS/MS in an appropriate manner for the period specified in the contract.

LSI Medience Corporation

1-13-4, Uchikanda, Chiyoda-ku, Tokyo 101-8517

## Study Sites and Investigators

A medical institution that can conduct the clinical study in compliance with GCP will be appointed as a study site. The investigator is appointed at each study site from the experts of the target disease of the investigational product or persons with equivalent experience.

The responsibilities of the investigator are as follows: to supervise the operations related to the clinical study and, if the clinical study is conducted by a team including subinvestigators and clinical research coordinators, to control and guide the team as a leader.

Study sites and investigators are listed in Attachment 1.

# LIST OF SUPPORTING DATA

Supporting Data 1: Standards for Classification of Seriousness of Adverse Drug Reactions by Drugs etc.

# LIST OF ATTACHMENTS

Attachment 1: Study sites and investigators

# REFERENCES

1. Veronese M. E., Mackenzie P.I. etc. Tolbutamide and phenytoin hydroxylations by cDNA-expressed human liver cytochrome P450 2C9. Biochem Biophys Res Commun 1991; 175: 1112-8.
2. Bajpai M. Roles of Cytochrome P4502C9 and Cytochrome P4502C19 in the Stereoselective Metabolism of Phenytoin to Its Major Metabolite (Short communication). Drug metabolism and disposition. 1996; 24: 1401-3.
3. Faucette S.R. Regulation of CYP2B6 in primary human hepatocytes by prototypical inducers. Drugs metabolism and disposition 2004;32.:348-58
4. Hatangndi V.S., Boas R.A. and Richards E.G. Postherpetic neuralgia : management with antiepileptic and tricyclic drugs. Advances in pain research therapy 1976. 583-7.
5. Braham J. and Saia A. Phenytoin in the treatment of trigeminal and other neuralgias. Lancet 1960; II:892-893.
6. Herpes Zoster and Varicella: Clinical Practice Strategy in the Era of Prevention[in Japanese], 1st Edition, Medical Tribune, 2016, pp. 101 to 05.
7. Herpes Zoster and Varicella: Clinical Practice Strategy in the Era of Prevention[in Japanese], 1st Edition, Medical Tribune, 2016, pp. 117 to 27.
8. Supporting Data for Application for Marketing Approval of Fostoin 750 mg for Injection: Summary Technical Documentation, Part 2 (M2.6.2.2 Primary Pharmacodynamics)

(http://www.pmda.go.jp/drugs/2011/P201100120/index.html)

1. McNamara J. Goodman and Gilman's "The Pharmacological Basis of Therapeutics." 2006; Ver. 11. P.501-525.
2. McLean M.J. and Macdonald R.L. Multiple actions of phenytoin on mouse spinal cord neurons in cell culture. J. Pharmacol. Exp. Ther 1983; 227: 779-789.
3. Fujii H, Fukushima T, Ishii M, Nagano Y, Kawanishi S, Watanabe Y, Kosogabe Y, Kagiki H, and Tokioka H: The efficacy of intravenous lidocaine for acute herpetic pain—placebo controlled trial. Masui, 2009, p. 1413 to 1417
4. Attal N Intravenous lidocaine in central pain: a double-blind, placebo-controlled, psychophysical study. Neurology. 2000 Feb 8;54(3):564-74.
5. Salas S, Auquier P, Duffaud F, Garnier SR, Deschamps M, Honoré S, Sudour P, Baumstarck K. Efficacy of lidocaine in patients receiving palliative care with opioid-refractory cancer pain with a neuropathic component: study protocol for a randomized controlled study. Trials. 2014 Aug 12;15:318. doi: 10.1186/1745-6215-15-318.
6. Manuals for Management of Individual Serious Adverse Drug Reactions by the Ministry of Health, Labour and Welfare (http://www.info.pmda.go.jp/juutoku/file/jfm1003012.pdf)
7. Motonobu Ito, Single-word Intelligibility Test for Evaluating Speech of Adults with Articulation Disorders. The Japan Journal of Logopedics and Phoniatrics 1992; 33: 227-36.
